# Leaf-FISH: Microscale Imaging of Bacterial Taxa on Phyllosphere

**DOI:** 10.3389/fmicb.2017.02669

**Published:** 2018-01-09

**Authors:** Elena L. Peredo, Sheri L. Simmons

**Affiliations:** Marine Biological Laboratory, Josephine Bay Paul Center, Woods Hole, MA, United States

**Keywords:** *Arabidopsis thaliana*, combinatorial labeling, fluorescence *in situ* hybridization, leaf microenvironments, *Methylobacterium*, phyllosphere, preferential colonization, *Zostera marina*

## Abstract

Molecular methods for microbial community characterization have uncovered environmental and plant-associated factors shaping phyllosphere communities. Variables undetectable using bulk methods can play an important role in shaping plant-microbe interactions. Microscale analysis of bacterial dynamics in the phyllosphere requires imaging techniques specially adapted to the high autoflouresence and 3-D structure of the leaf surface. We present an easily-transferable method (Leaf-FISH) to generate high-resolution tridimensional images of leaf surfaces that allows simultaneous visualization of multiple bacterial taxa in a structurally informed context, using taxon-specific fluorescently labeled oligonucleotide probes. Using a combination of leaf pretreatments coupled with spectral imaging confocal microscopy, we demonstrate the successful imaging bacterial taxa at the genus level on cuticular and subcuticular leaf areas. Our results confirm that different bacterial species, including closely related isolates, colonize distinct microhabitats in the leaf. We demonstrate that highly related *Methylobacterium* species have distinct colonization patterns that could not be predicted by shared physiological traits, such as carbon source requirements or phytohormone production. High-resolution characterization of microbial colonization patterns is critical for an accurate understanding of microbe-microbe and microbe-plant interactions, and for the development of foliar bacteria as plant-protective agents.

## Introduction

Plants harbor diverse microbial communities composed of yeast, fungi, archaea, bacteria and viruses. Clear differences between plant microbiomes and environmental inocula (air, Maignien et al., 2014, and soil Lundberg et al., 2012) indicate that the plant habitat enriches for particular types of microbes. Microbial communities are also clearly differentiated by plant genotype (Bodenhausen et al., 2014; Horton et al., 2014) and plant organ (Knief et al., 2012; Bodenhausen et al., 2013). Plants partially control microbial colonization processes by modifying the composition of nutrients in the local environment (Bulgarelli et al., 2013). The plant immune system responds to pathogenic bacteria with a variety of strategies that range from programmed death to the release of antimicrobial compounds, while allowing colonization by beneficial and commensal bacteria (Lebeis, 2014).

Aerial plant tissues are characterized by the presence of a waxy substance generated by the epidermis, the cuticle. With water permeability nearly three orders of magnitude lower than plant cell walls, the cuticle protects leaves against desiccation, radiation and pathogenic agents (Domínguez et al., 2011). Despite these harsh conditions, the phyllosphere harbors a diverse microbial community with estimated densities of 10^6^-10^7^ cells cm^−2^ (Lindow and Brandl, 2003), which is shaped by host plant genotype (Knief et al., 2010b; Redford et al., 2010; Horton et al., 2014), seasonal changes (Ercolani, 1991), and geography (Knief et al., 2010b). Phyllosphere microorganisms display specialized adaptive traits to the extremely dry and oligotrophic ephemeral environments on leaf surfaces such as production of phytohormones, antibiotics and pigments, aggregation, and chemotaxis (Vorholt, 2012). In particular, auxin biosynthesis is common among plant-associated bacteria in the phyllosphere (Lindow and Brandl, 2003), has known plant-growth promoting effects, and is thought to facilitate infection by leaf pathogens (reviewed in Spaepen and Vanderleyden, 2011).

Culture-independent molecular techniques have been used to characterize phyllosphere microbial diversity in an increasing number of plant species (e.g., Lambais et al., 2006; Knief et al., 2008, 2010b; Delmotte et al., 2009; Redford et al., 2010; Maignien et al., 2014; Copeland et al., 2015) but our understanding of phyllosphere microbial dynamics is still limited. Phyllosphere communities typically contain hundreds of microbial taxa (Ottesen et al., 2013; Maignien et al., 2014), roughly an order of magnitude less than rhizosphere assemblages. Phyllosphere microbial characterization is frequently achieved by high-throughput sequencing of phylogenetic marker genes, including 16S rRNA in bacteria (Lundberg et al., 2012; Maignien et al., 2014) and ITS in fungi (Cordier et al., 2012). Relatively few studies have examined the functional profiles of phyllosphere communities (Delmotte et al., 2009; Atamna-Ismaeel et al., 2012; Knief et al., 2012; Ottesen et al., 2013; Chafee et al., 2015). Bulk-leaf sequencing methods, however, erase evidence of the microscale interactions between bacteria and the leaf surface (Cardinale, 2014; Esser et al., 2014). Phyllosphere taxa overcome the extreme environmental conditions on leaf surfaces (UV, desiccation) in part by colonizing a variety of heterogeneous microhabitats with favorable conditions for epiphytic bacteria (Melotto et al., 2008). Cuticle cracks, stomata, hydathodes, veins, trichomes, and glands are important in bacterial colonization and play a key role in colonization of leaves by human pathogens, an important issue for food safety (Teplitski et al., 2011). For example, the pathogen *Clavibacter michiganensis* colonized tomato leaves via hydathodes (Carlton et al., 1998), *Salmonella enterica* preferentially colonized trichomes on tomato leaves (Barak et al., 2011), and cells of *E. coli* O157:H7 that penetrated stomata resisted chlorine treatment more effectively (Saldaña et al., 2011).

In order to investigate the dynamics of bacterial colonization and bacterial-host interactions in the phyllosphere we need techniques to visualize microbial distributions at high resolution on the microscale level. Genetically altered bacteria expressing fluorescence marker genes such as GFP have revealed some key processes of phyllosphere colonization. These include evidence for aggregate formation (Monier and Lindow, 2003, 2005b), survival rates of immigrant bacteria (Monier and Lindow, 2005a), changes in metabolic rates (Leveau and Lindow, 2001), colonization patterns (Compant and Reiter, 2005; Barak et al., 2011), quorum size (Dulla and Lindow, 2008), and reproductive success (Remus-Emsermann and Leveau, 2010). Naturally occurring leaf bacteria have been visualized using non-specific dyes coupled with scanning electron microscopy, epifluorescence microscopy and confocal microscopy (Davis and Brlansky, 1991; Morris et al., 1997; Fett and Cooke, 2003). Due to high autofluorescence, leaves are a challenging surface for fluorescence *in situ* hybridization (FISH) studies using oligonucleotide probes designed to target specific bacterial taxa. There are few examples of FISH protocols optimized for visualization of microbial distributions on aerial plant tissue (Li et al., 1997; Brandl et al., 2001; Shinkai and Kobayashi, 2007; Piccolo et al., 2010). An elegant bypass for plant autofluorescence has been the use of TAPE-FISH, transference of surface microorganisms to an adhesive tape, used in tomato fruits (Bisha and Brehm-Stecher, 2009) and in leaves of *Arabidopsis thaliana* (Remus-Emsermann et al., 2014).

In this work, we describe a robust and easily transferable protocol to generate *in planta* tridimensional images for visualization of multispecies epiphytic microbial communities of leaves using *Arabidopsis thaliana* as a model organism. The advantage of our Leaf-FISH method over prior work is the ability to visualize multiple taxonomically distinct microbes directly in association with leaf microstructures. Our method builds on of combinatorial labeling and spectral imaging fluorescent *in situ* hybridization method (CLASI-FISH) (Valm et al., 2011, 2012). CLASI-FISH uses spectral deconvolution to separate overlapping fluorescence signals from multiple distinct probes, allowing the simultaneous *in situ* visualization of an unlimited number of microbial taxa. We applied the same the principles to separate the specific fluorescence signal of the labeled probes targeting the bacteria in the phyllosphere to that from the plant tissue. Our method overcomes, and even takes advantage of, plant autofluorescence by using a simple and cost-effective pretreatment prior to tissue fixation, combined with confocal laser scanning microscopy (CLSM) and the principles of image processing of CLASI-FISH (Valm et al., 2011, 2012).

We used a multi-step approach to method validation. First, we defined fixation and hybridization methods using *in vitro* grown *Arabidopsis* inoculated with individual isolates. We used the validated method to examine the naturally occurring microbial distributions on leaves of greenhouse-grown *Arabidopsis*. We also tested the transferability of the method to other plant systems besides *Arabidopsis* using eelgrass, a submerged monocot highly adapted to oceanic lifestyle. Second, we demonstrated the suitability of Leaf-FISH for *in situ* taxonomical identification of leaf microorganisms by simultaneous detection of three phylogenetically diverse microbes *in situ* on *Arabidopsis* leaves. As a test case for the method, we investigated the spatial distributions of a reference and a newly isolated strain of the common pink-pigmented phyllosphere genus *Methylobacterium. A priori* we expected these strains to have similar distributions on the leaf surface due to their shared metabolic niche (facultative methylotrophy) and ability to produce active phytohormones. Imaging indicated, however, that even closely-related bacterial species sharing common physiological traits can consistently occupy extremely different leaf microniches. This test case illustrates the difficulty of predicting bacterial distributions in the phyllosphere based solely on phylogenetic and physiological information and reaffirms the importance of developing imaging methods suitable for *in situ* study of phyllosphere microbiome.

## Materials and methods

### Plant material

#### Arabidopsis

Two different sources of leaves were used in this study: greenhouse grown *A. thaliana* L. plants cvs. Columbia (Col-0) (Lehle Seeds, USA), naturally colonized by environmental bacteria (see Maignien et al., 2014 for details) and *in vitro* grown plants (0.5 X MS with vitamins at pH 5.8 with 0.7% agar, Figure S1A) grown from surface sterilized seeds. Leaves excised from axenic plants incubated overnight with defined mixtures of bacteria were imaged to showcase the suitability of Leaf-FISH for taxa identification (see section Visualization of Multiple Bacterial Taxa in Arabidopsis Leaves for full description of incubation methods). Leaves from plants grown from seeds inoculated with *Methylobacterium adhaesivum* B5A (isolated in this study, see Methods on Supplementary Material) or the reference strain *Methylobacterium extorquens* PA1 (Knief et al., 2010a) were used to explore leaf colonization preferences under *in vitro* conditions (see sections Monoisolate Inoculation of Arabidopsis Seeds and Characterization of *Methylobacterium* strains for colonization of *Arabidopsis* phyllosphere for full description of inoculation methods).

#### Zostera

Eelgrass (*Zostera marina*) was collected in Woods Hole, MA (US) in June 2013.

### FISH and Leaf-FISH method

#### Fixation

Fresh leaves (10–20 *in vitro* leaves/leaf fragments per tube) (Figure S1B) were incubated at room temperature on an orbital shaker at low speed (90 rpm) for 1 h in 10 ml of 1 X PBS (Fisher Scientific, USA) supplemented with 2% paraformaldehyde (EMS, USA). We tested other technical approaches to optimize the fixation step, such as variations of the agar embedding technique described in Brandl et al. (2001). Specifically, we embedded leaf sections in 0.8% low melt agarose using small hybridization chambers (e.g., Secure-Seal, EMS, USA). However, the use of agarose was extremely labor intensive and agarose coated-leaves were labile and difficult to manage. We combined agarose coating with a light vacuum to increase the effectiveness of the fixation step but this resulted in in severe alteration of the distribution of the bacterial cells, many of which were removed from the plant tissues and found in the agarose coat. Processing of “naked” leaves, defined here as tissues not embedded or treated with any coating agents prior to fixation, required less intensive manipulation and reduced preparation time and costs, as it allowed pooling leaves during fixation and pigment removal steps. The lack of protective coating agent did not result in significant losses of bacteria from leaf surfaces (see Supplementary Material).

#### Pigment removal

Leaves were incubated in 10 ml of freshly prepared 50% ethanol. Sequentially, the percentage of ethanol in the mixture was increased (70, 80, 85, 90, and 95%) and, in each step, leaves were incubated for at least 30 min. Typically, *in vitro* material was incubated twice in 95% ethanol while greenhouse material required three to five replacements to achieve complete whitening of the tissues (Figures S1C, S2A,B). Plant material was stored at −20°C in 10 ml of 95% ethanol for 2 days prior to further optimize pigment removal. Long-term storage of fixed tissues was performed at −20°C in 50% ethanol/1 X PBS.

#### Hybridization

Tissues were rehydrated prior to any hybridization steps in sterile distilled water or 1 X PBS. Two to four leaves were transferred to each 0.7 ml Eppendorf tube containing 400 μl of pre-warmed hybridization mix and incubated at 46°C for 1.5 h. Hybridization buffer (0.01% SDS, 0.02 M Tris-HCl pH 7.5, 0.2 M/0.1 M NaCl and 20%/30% “Hi-Di” Formamide) was prepared fresh. Appropriated probe(s) combinations were added to each hybridization mix for a final concentration of 5 ng μl^−1^ each. The probes used in this study were the general eubacteria set EUB338 I, II, and III (Amann et al., 1990; Daims et al., 1999), *Methylobacterium*-specific mybm-1388 (Pirttilä et al., 2000) and *Pseudomonas*-specific PSE227 (Watt et al., 2006). Leaves were transferred to new tubes with 1 ml of pre-warmed wash buffer (0.01% SDS, 0.02 M Tris-HCl pH 8.0. 0.22/0.11 M NaCl; 5 mM EDTA if stringency ≥20%) and incubated at 48°C for 10 min. Leaves were rinsed with sterile distilled water, placed on a microscopy slide and tissues were dried in the dark. ProLongGold with or without DAPI (Life Technologies, USA) was added to each leaf before covering with a glass cover. Samples were stored overnight in the dark at room temperature. For long-term storage, slides were kept at 4°C.

#### Probe specificity testing and evaluation

The hybridization specificity of all probes was tested by evaluating their performance in pure and mixed cultures of bacteria. An appropriate volume of bacterial culture was filtered through a 0.22 μm filter (GTTP, Millipore, USA). After fixation in 1 X PBS and 1% paraformaldehyde for 1 h at room temperature, sections of the filters were subjected to a standard *in situ* hybridization protocol using a mixture of probe EUB338 I, II, and III conjugated with Rhodamine Red, mybm-1388 conjugated with Alexa-488 and PSE227 conjugated with Alexa-647 (Life Technologies, USA). Total number of cells present per field were evaluated by counting in a standard epifluorescence microscope (Zeiss Axioskop2) using DAPI staining.

#### Image acquisition and analysis

Images were acquired with a Zeiss LSM 780 (Carl Zeiss Microscopy GmbH, Germany) laser scanning confocal microscope equipped with 32-channel GaAsP detector and 8 laser lines using a 100 × 1.4 NA objective lens. Each field of view was imaged using sequential excitation with the appropriate laser combinations for each fluorochrome combination, including 633- (Alexa-647, emission ~670 nm), 561- (Rhodamine Red, emission ~591 nm), and 488- (Alexa-488, emission ~520 nm). To maximize the signal-noise ratio each image was obtained as the average of four images, at a resolution of 12 or 16-bit (4,096–65,536 gray levels) and frame size of 1024 X 1024. The resulting size of the images was 84.94 μm × 84.94 μm. 3-D images were generated using Z-stacks in multitrack mode. The number of tracks was optimized using the multitracking option to minimize cross-talk and fluorophores bleaching. To generate 3-D images typically 5–16 images were taken per field, each at 0.5–1 μm across the Z-axis. Starting from the leaf surface, our ability to image the leaf inner tissues was determined by the residual background autofluorescence emitted by the leaf cells. Subcuticle and substomata areas were easily imaged (approx. 10–15 μm depth), even in mature leaves from greenhouse plants. In young leaves from *in vitro* plants, the low autofluorescence after pigment removal made possible to image across the blade. Larger composite images, up to 400 μm × 400 μm, were generated by tiling smaller ones generated as described above. Capture in lambda mode was used to generate crosstalk-free multi-fluorescence images by parallel or sequential acquisition of images with spectral information of each pixel.

Spectral images were unmixed using the linear unmixing algorithm implemented in the ZEN Software package (Carl Zeiss) following manufacturer protocols. Linear unmixing was applied using appropriate reference spectra library acquired defining region of interest (ROI) in sets of reference images. For identification of background plant-associated autofluorescence multiple samples of plant tissues (surface cuticle areas, trichomes, chloroplasts, and certain areas of stomata) were included in the reference spectra libraries. ROI were drawn on images corresponding different *Arabidopsis* leaves and included samples collected from different structures and depths. In most cases a combination of three plant associated references were enough to unmix images and minimize the signal in the residual component. Reference spectra also included unlabeled cells grown as monocultures of *Methylobacterium, Sphingomonas*, and *Pseudomonas* for detection of possible autofluorescence as that shown for *Pseudomonas* in Video S2; cells in culture of *Sphingomonas* labeled with EUB338 (Rhodamine Red), *Methylobacterium* labeled EUB338 (Rhodamine Red) and mybm-1388 (Alexa-488) and *Pseudomonas* labeled with EUB338 (Rhodamine Red) and PSE227 (Alexa-647) and acquired with each excitation wave length. For multicolored Leaf-FISH image presentation, the image of each unmixed fluorophore channel was assigned a different pseudocolor. As recommended in Valm et al. (2012), autofluorescence layers were treated as other spectral character to be identified after unmixing. To generate the multicolor Leaf-FISH, images generated with each unmixed fluorochrome plus those generated with the unmixed autofluorescence of the plant tissues were false colored. All resultant images were merged, and brightness was independently adjusted to generate the final image. Additional imaging processing was performed in Fiji (Schindelin et al., 2012).

### Visualization of multiple bacterial taxa in *Arabidopsis* leaves

We tested the performance of Leaf-FISH method for bacterial taxonomical identification using sterile leaves from *in vitro* grown *Arabidopsis* incubated in a known mixture of bacterial isolates. These included *Sphingomonas* sp. (C3A2) and *Methylobacterium adhaesivum* (B5A) (isolated in this study; see Supplementary Material), and the poplar endophyte *Pseudomonas putida* W619 (Taghavi et al., 2009) kindly provided by Dr. Daniel van der Lelie. Prior inoculation all isolates were cultured at 30°C and 180 rpm for 48 h in appropriated liquid medium, LB (0.5 g l^−1^ NaCl). Cell density and viability of the cultures was assessed using Live/Dead BacLight L7012 (Live Technologies, USA). An aliquot of each culture was added to a 50 mL Falcon tube containing 15–20 *Arabidopsis* leaves freshly excised and 30 ml of fresh LB and incubated overnight as described above. To compensate for the fast growth of *Pseudomonas* on LB medium, 10 times more of the *Methylobacterium* and *Sphingomonas* cultures were added to the incubation mix (1 ml vs. 100 μl of *Pseudomonas*). Prior to fixation and hybridization for bacterial visualization, leaves were rinsed in sterile distilled water to remove bacterial cells not attached to leaf surfaces.

### Colonization of *Arabidopsis* phyllosphere under *in Vitro* conditions

#### Monoisolate inoculation of arabidopsis seeds

*Arabidopsis* sterile seeds (10% bleach 20 min, rinsed in sterile distilled water 10 times) were incubated for 20 min at room temperature in 1.5 ml Eppendorf tubes containing 0.5 ml of 10^4^ cell ml^−1^ of the appropriate bacterium isolate. Each bacterial isolate used for seed inoculation were cultured at 30°C and 180 rpm for 48 h in liquid medium, LB (0.5 g l^−1^ NaCl) or MM medium. Prior inoculation, cell density and viability of the culture was assessed using Live/Dead BacLight L7012 (Live Technologies, USA). Sterile and inoculated seeds were transferred to respective rectangular transparent polypropylene microboxes (Combiness Microbox, Belgium) (10–12 seeds per microbox, four microboxes per treatment per experiment). Plants were grown in 200 ml of autoclaved 0.5 X minimal MS medium with vitamins, at pH 5.8, 0.7% agar and filtered lids (+XXL). Previous experiments concluded that high gas exchange (81.35 GE day^−1^) contributed to general macroscopic features of the leaves and more vigorous growth in carbon source-free MS medium (data not shown). Sowed seeds were stored at 4°C in the dark for a period of 48–72 h. Germination was carried out under low light, achieved by covering the microboxes with a shadow mesh in a Precision Plant Growth Refrigerated Incubator 818 (Thermo Scientific, United States) under 8 h light (23°C) 16 h dark (21°C) cycle. After 2 weeks, seedlings were exposed to full light conditions (120–180 μmol m^−2^ s^−1^). Microboxes were rotated each 2–3 days to minimize positional effects. Adult stage was reached after 6 weeks in culture (bolting observed in less than 25% of plants).

For this seed inoculation protocol, we defined as “successful colonizer” any isolate that consistently demonstrated abundant colonization of *Arabidopsis* leaves without interfering with the development of the host plant. In addition, we consider as a desirable trait low growth on the MS medium plant medium. Following the criteria described above, we selected for colonization of *Arabidopsis* phyllosphere under *in vitro* condition studies two strains of *Methylobacterium; M. adhaesivum* (B5A) isolated in this study and a closely-related laboratory-evolved isolate of *M. extorquens* PA1. *In vitro* plants grown from inoculated seeds were then used for Leaf-FISH experiments. Sterile plants were grown as reference and used to evaluate any potential alterations of plant morphology or development caused by the seed inoculation. To assure the repeatability of the colonization patterns observed, two cohorts of plants were independently seed-inoculated for each isolate of *Methylobacterium*.

#### Characterization of *Methylobacterium* strains for colonization of *Arabidopsis* phyllosphere

Indolic acid (IAA) production of *Methylobacterium adhaesivum* B5A and the reference strain *Methylobacterium extorquens* PA1 was measured as defined in Glickmann and Dessaux (1995). Phytohormone production was assessed by triplicate on cultures grown in liquid LB and MM at 30°C and 180 rpm for a period of 10 days. The second, seventh and tenth day in culture, optical density at 600 nm and IAA concentration (Salkowski's reagent R2, 4.5 g of FeCl_3_ per liter in 10.8 M of H_2_SO_4_) at 535 nm were measured. Indol concentration was determined using a five point concentration curve (0-10 mg ml^−1^) of synthetic 3-indolacetic acid (Sigma-Aldrich, USA) freshly prepared in each respective medium. The *in vivo* effect of IAA production of each *Methylobacterium* strain (B5A, PA1) was evaluated by quantification of their ability to trigger an adventitious organogenic response of *Arabidopsis* leaf tissues. Leaves freshly excised from several 6-week-old *in vitro Arabidopsis* axenic plants were pooled and incubated at room temperature for 20 min in saline double distilled water (0.85% NaCl) or saline water supplemented with an aliquot of *Methylobacterium* (strains B5A or PA1) grown 48 h in MM. Rinsed leaves (8–13) were placed in petri dishes (eight plates per strain) with 10 ml of MS with vitamins pH 5.8, 0.7% agar supplemented with 30 g l^−1^ of sugar and maintained under an 8 h light (120–180 μmol m^−2^ s^−1^) 16 h dark cycle. Control leaves (axenic) were placed in MS medium (negative controls, triplicate) or MS medium supplemented with 1.5 mg l^−1^ IAA (positive controls, triplicate). The presence of adventitious roots or calli was determined every 2 weeks by visual inspection over a 6-week period.

## Results

**Figure 1:**
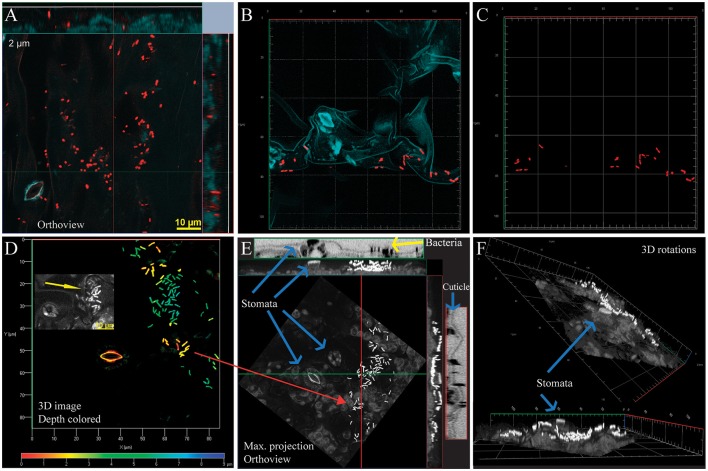
Bacteria on the surface of greenhouse grown *Arabidopsis* associate strongly with leaf microstructures. All bacterial cells were labeled with the general probe EUB338 I, II, and III (here and elsewhere probe shown in red unless otherwise specified). All images were captured using a 100 X lens on a confocal microscope Zeiss LSM780. **(A)** Orthogonal projection, 13 confocal images. Bacteria on leaf surface distribute along the irregular profile of the leaf surface, as shown by the leaf autofluorescence (blue, here and elsewhere). **(B)** 3-D composite image using Z-stack capture, 6 images (5 μm depth), and analyzed using linear unmixing. **(C)** Same image as **(B)**, background removed to facilitate visualization of bacteria. **(D–F)** Different views of the same image generated using Z-stack capture, 19 images (9 μm depth). **(D)** 3-D rendering image. The image is color-coded to indicate distance from the surface (red to dark blue). Inset shows high contrast detail to illustrate the cuticle irregularities in that area (yellow arrow). **(E)** Maximum projection orthoview (X, Y, and Z) showing bacteria on cuticle. Plant autofluorescence is shown in gray, bacteria in white. In the outside, orthoviews generated in Fiji from fluorescence data showcasing surface irregularities (blue arrows). Bacteria are shown in black (yellow arrow) **(F)** 3D renderings of same image. Further information can be found in Video S1 (fluorescence from the EUB338, transmitted light captured during confocal scanning microscopy, and overlap of both signals). Yellow bar, 10 μm.

**Figure 2:**
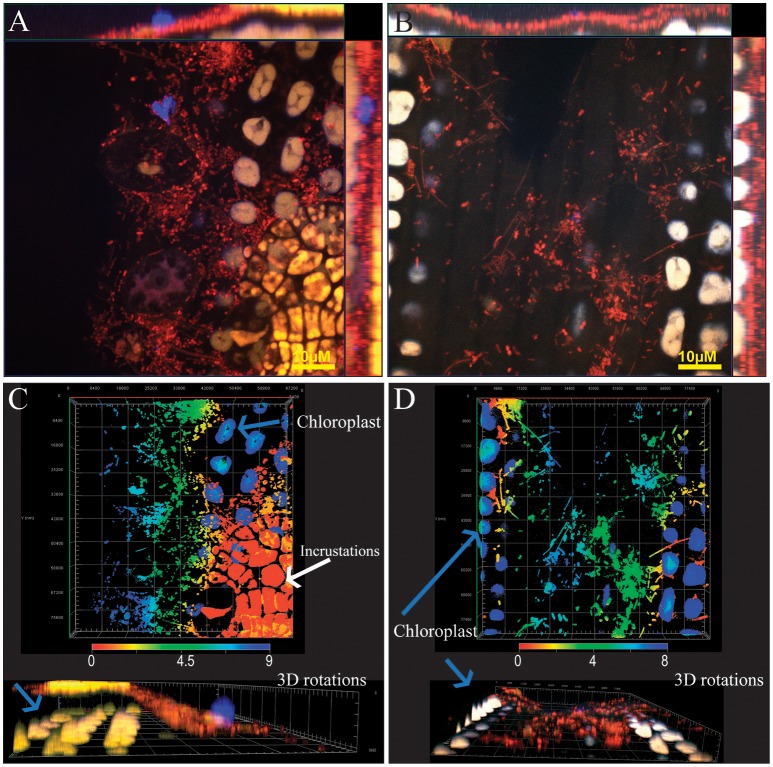
Visualization of bacteria on leaves of the aquatic angiosperm *Zostera marina* (eelgrass). All bacterial cells were labeled with the general probe EUB338 I, II, and III (red). All images were captured using a 100 X lens on a confocal microscope Zeiss LSM780. **(A,B)** Maximum projection orthoview (X, Y, and Z) of Z-stack images (10 and 9) captured in channel mode, separated 1 μm each (100 X lens, Zeiss LSM780). High density of bacterial cells can be observed on the leaf surface. **(C,D)** 3-D composite images generated using Z-stack capture, (9 and 8 μm depth). The images are color-coded to indicate distance from the surface (red to dark blue). 3-D views from Z axis are also provided. Colonization of the subcuticle area was not observed. Yellow bar, 10 μm.

**Figure 3:**
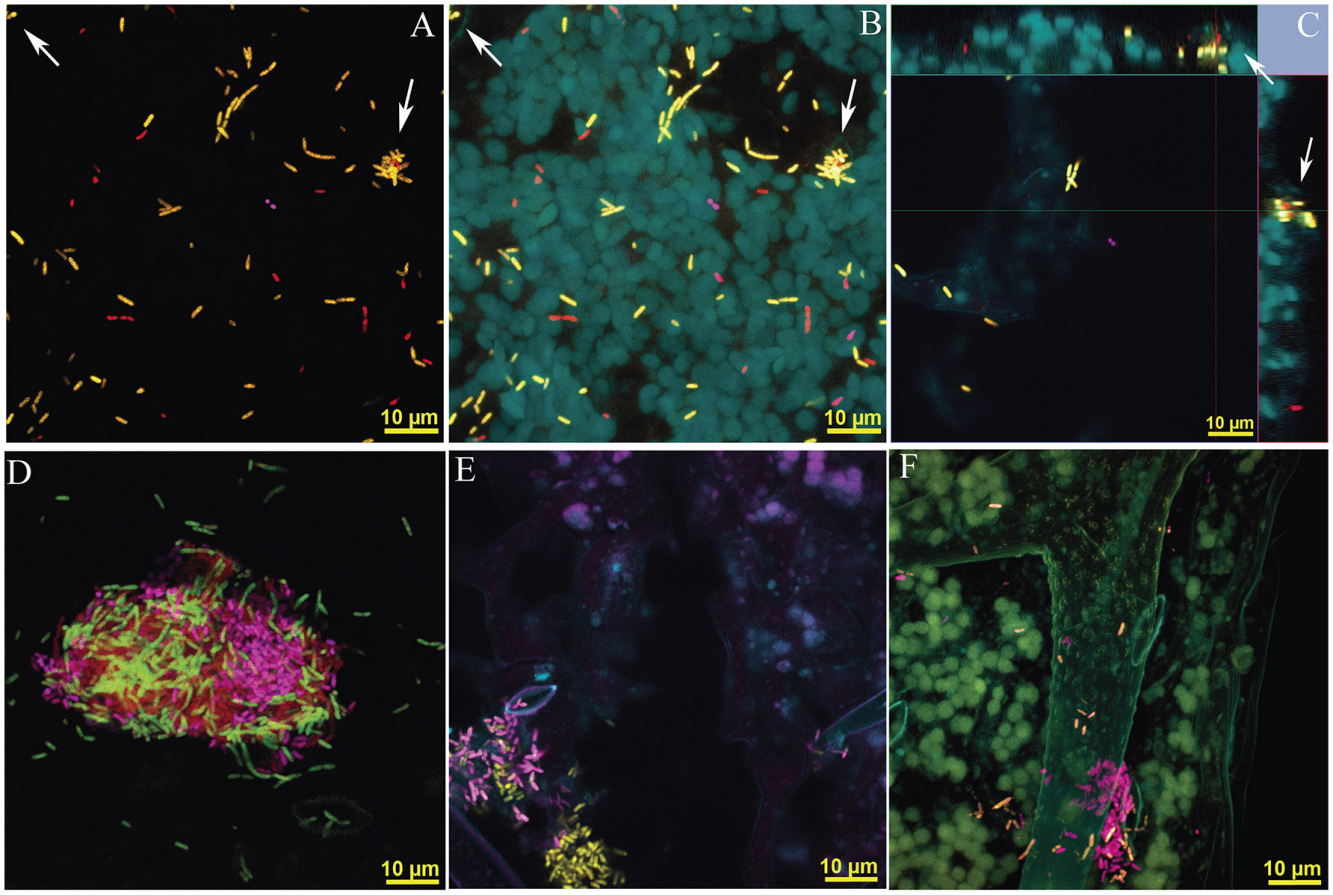
Imaging of mixed microbial aggregates on the leaf surface and immediate subcuticle area of *in vitro* grown *Arabidopsis* leaves incubated with *Sphingomonas, Methylobacterium* and *Pseudomonas*. All confocal images were generated using a 100 X lens, Zeiss LSM780 and processed using spectral unmixing. *Methylobacterium* and *Pseudomomas* are labeled with a combination of probes (EUB338 I, II, and III and mybm-1388 or PSE227), and are false colored in pink and yellow respectively. *Sphingomonas* is only labeled with EUB338 I, II and III and shown in red. FISH EUB338 I, II, and III conjugated with rhodamine, mybm-1388 with Alexa-488, and PSE227 with Alexa-647. **(A–C)** Correspond to different processing of an Z-stack image, 16 confocal images, 1 μm apart. Stomatas are signaled with arrows **(A)** 3-D image generated using only layers identified as specific fluorescence emission of general and taxa-specific bacterial probe(s) (Figure S5 for fluorophore specific decomposition). In plate **(B)** plant autofluorescence is incorporated as an additional layer to the 3D image **(C)** Ortho projection of Z-stack images, 15 μm depth. Bacteria are distributed in peaks and groves of the plant surface, so only a few cells are visible in a single plane. Arrows signal cells inside the stomatic chamber. Main image corresponds to Z = 0 (see Video S3 in Supplementary Material) **(D)** Aggregate formed by cells identified as *Methylobacterium, Pseudomonas* and *Sphingomonas*. **(E)** Image showing *Pseudomonas* and *Methylobacterium adhaesivum* B5A near stomata. **(F)** *Pseudomonas* and *Methylobacterium adhaesivum* B5A forming aggregates on an *Arabidopsis* trichome, 15 Z-Stack images total depth of 14 μm. In all panels, yellow bar, 10 μm.

**Figure 4:**
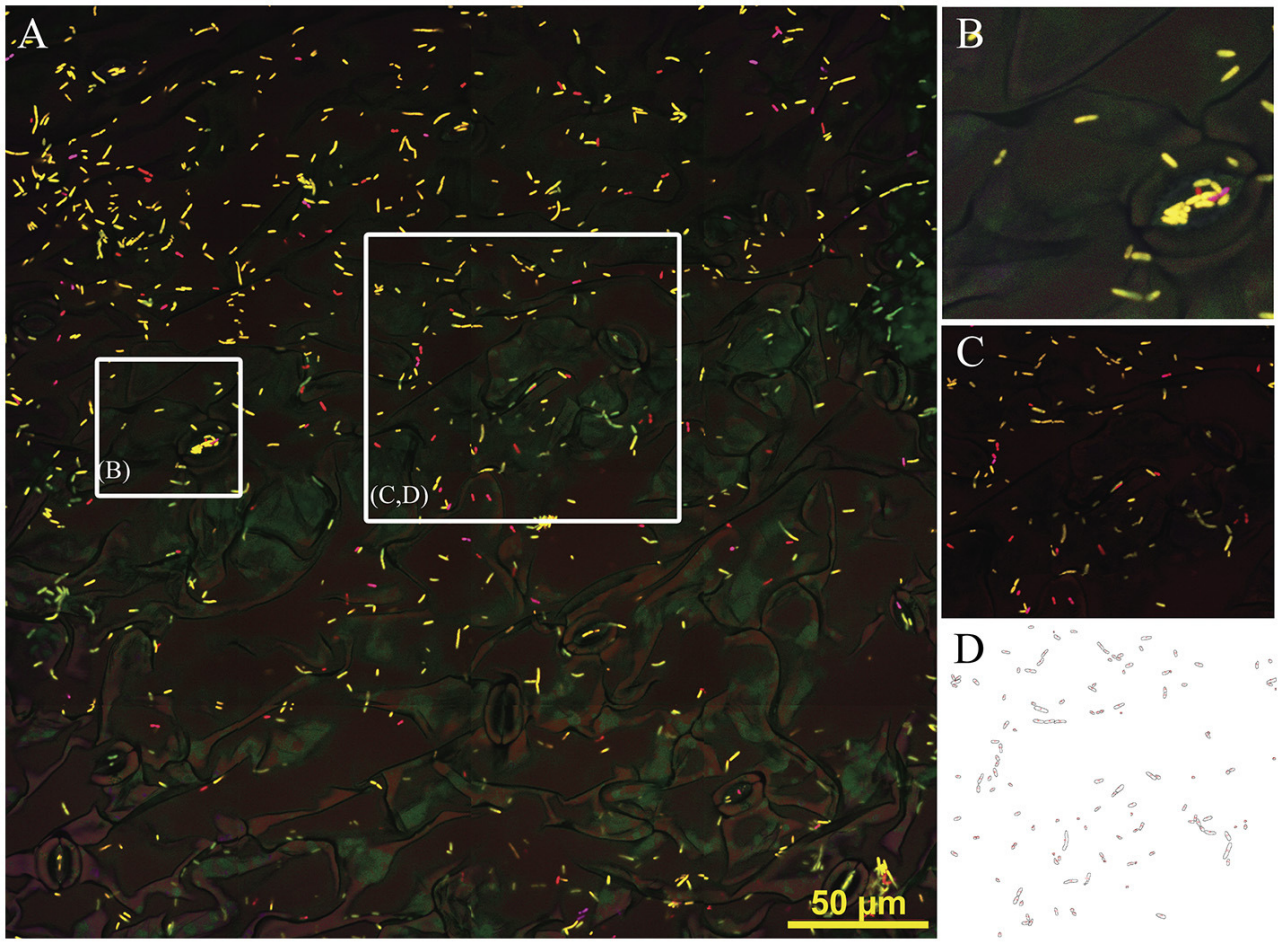
Composite 3-D image (4 × 4 tiles, approx. 340 μm) showing *Sphingomonas, Methylobacterium* and *Pseudomonas* on the leaf surface of *in vitro* grown *Arabidopsis*. **(A)** Hybridization mix included probes EUB338 I, II and III (conjugated with Rhodamine, false colored in red), *Methylobacterium* mybm-1388 (Alexa-488pink) and *Pseudomonas* PSE227 (Alexa-647, yellow). **(B)** Detail showing cells in stomata area, box approximate 50 μm. **(C)** Image after background removal, box ~100 μm. **(D)** Result of automatic particle counting, indicating the suitability of images for automatic cell quantification (Fiji). Yellow bar, 50 μm.

**Figure 5:**
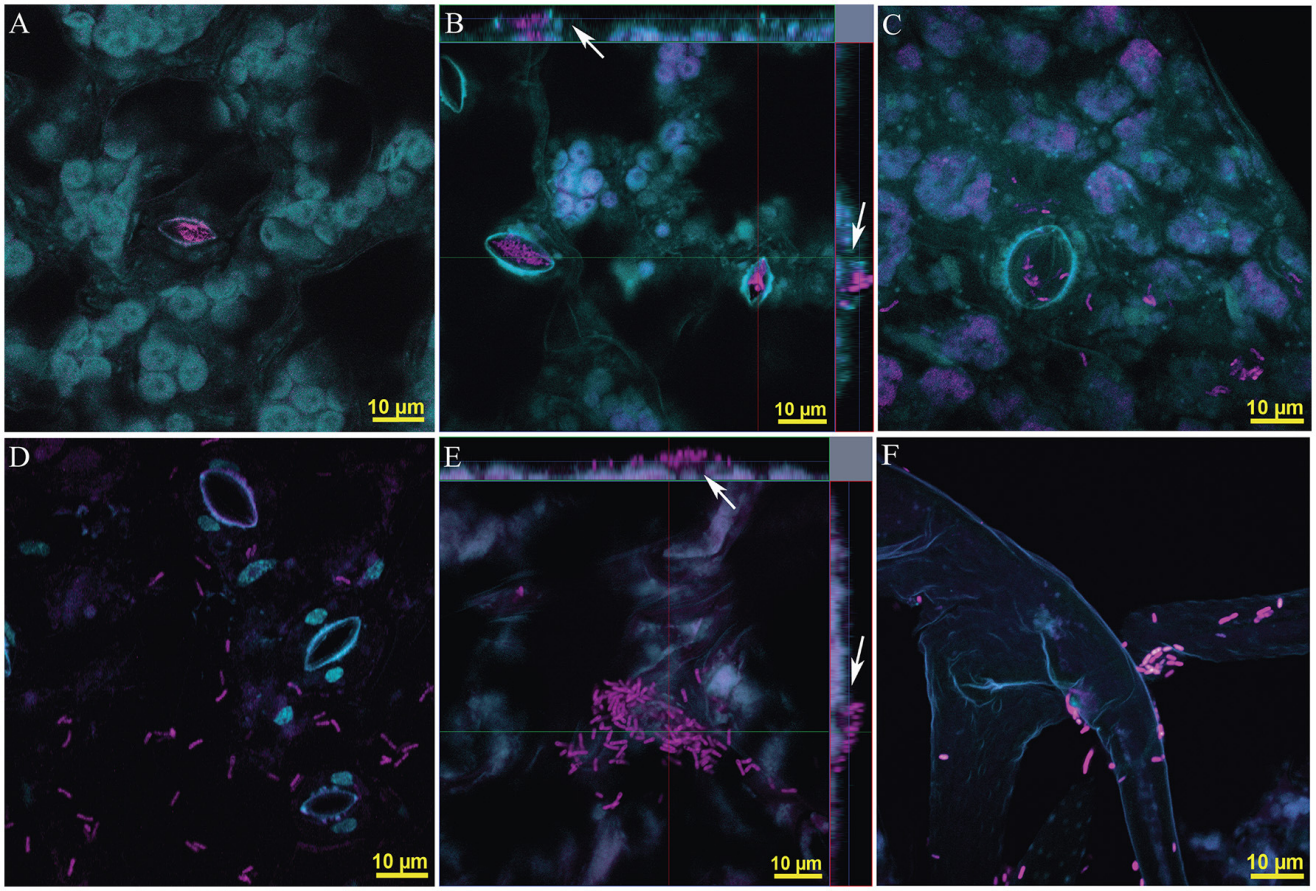
Preferential microhabitat of two *Methylobacterium* species in monoisolate seed inculated *in vitro* grown *Arabidopsis* (100 X lens, Zeiss LSM780). **(A–C)** *M. extorquens* PA1 was preferentially found in the stomatic chambers. **(A)** FISH EUB338 I, II and III conjugated with Alexa-488; **(B)** Ortho projection of Z-stack images, 8 μm depth, FISH EUB338 I, II, and III conjugated with rhodamine. **(C)** Maximum intensity projection, composed of four images (4 μm depth). **(D–F)** *M. adhaesivum* B5A preferentially inhabits leaf surface. **(D)** FISH EUB338 I, II, and III conjugated with Alexa-488; **(E)** Ortho projection of Z-stack images, 9 μm depth, FISH EUB338 I, II, and III conjugated with rhodamine and mybm-1388 conjugated with Alexa-488. **(F)** Maximum intensity projection, composed of five images (5 μm depth). *M. adhaesivum* B5A on plant trichome. To facilitate visualization, in each plate plant fluorescence was false colored in cyan and bacteria were false colored in pink. Yellow bar, 10 μm.

The FISH method described here generates high resolution images of phyllosphere microorganisms presented on leaf surfaces and subcuticle areas while preserving the structural context provided by the leaf microstructures (Figures 1–5). The key features of Leaf-FISH method are background autofluorescence reduction, and preservation of bacterial distribution and plant microstructures, while still achieving a strong, specific hybridization signal from bacterial cells. Reduction of background signal facilitates visualization using traditional FISH methods, even in epifluorescence microscopes (Figures S1F, S2B). The capture and image processing following the CLASI-FISH approach (Valm et al., 2012) allows discrimination of compound fluorescent signals from bacteria labeled with multiple probes and even autofluorescence, on the technically challenging surface of leaves (Figures 3, 4). Leaf-FISH is method is equally effective detecting bacteria on greenhouse (Figure 1) or *in vitro* (Figures 1, 3–5) grown *Arabidopsis* leaves. Additionally, our protocol is easily transferable to other plant species (Figure 2). We also demonstrate the application of our method to identifying microhabitat preferences in two strains of the genus *Methylobacterium* (Figure 5).

### Method validation: technical testing

#### Reduction in autofluorescence allows detailed 3-D visualization of bacterial distributions on leaf surfaces

Background signal caused by the autofluorescence of *Arabidopsis* leaves completely masked any signal emitted by the fluorophores of the bacterial probes (Figures S2C,D) using a routine microscope for transmitted light and incident light fluorescence (Zeiss Axioskop2). CLSM (Zeiss LSM780) followed by intensive processing of confocal images post-capture led to slightly better results in leaf margins (data not shown) but we were unable to confirm bacterial visualization of other leaf areas. In order to reduce the background fluorescence emitted by the plant tissues, we incorporated in our protocol a pretreatment step consisting of a series of dehydrating steps in ethanol. The use of ethanol to gradually dehydrate *Arabidopsis* leaves successfully removes most of the pigments from the cells (Figure S1C). Under microscope, treated leaves show a translucent (bleached) appearance (Figure S2B) and lower levels of autofluorescence. Background signal reduction permitted clear identification of the specific fluorescence emission of the bacterial probes and visualization of the bacterial cells present on leaves (Figures S2E–H) even using routine microscopes (Zeiss Axioskop2).

In images generated through spectral imaging confocal microscopy, plant residual autofluorescence was incorporated into confocal images after unmixing as layer(s) that could be modified during post-imaging processing. Background signal(s) generated detailed images of the plant surface and subcuticular areas, allowing simultaneous visualization of the leaf microstructures and associated bacteria (Figures 1A–C). Tridimensional images show the continuous distribution of some bacteria on the leaf surface into subcuticle tissues (Figure 1A). Each of the probe fluorophores and plant autofluorescence were incorporated to the image as an individual layer that can be shown simultaneously (Figure 1B) or independently (Figure 1C) as 2-D or 3-D images. As expected, bacterial cells are irregularly distributed on peak and valleys of the leaf surfaces (Figures 1D,E) and are frequently found associated with cuticle cracks, stomatas, or close to veins.

#### Transferability of the Leaf-FISH method to other plant systems

We selected eelgrass (*Zostera marina*) to test the transferability of the Leaf-FISH method to other plant systems. Eelgrass's adaptations to oceanic lifestyle affect crucial mechanisms that terrestrial plants use to influence the structure of their associated leaf microbiomes. Eelgrass lacks stomata (a common entry point for pathogens in land plants), lost genes for the production of volatile terpenes, and presents a modified cell wall with low-methylated pectins and sulfated galactans, important for ion homoeostasis, nutrient uptake and O_2_/CO_2_ in saline conditions (Olsen et al., 2016). Land and aquatic plants also differ in leaf microbiome core composition and dynamics. While leaf microbiome is distinct from air inoculum in *Arabidopsis* (Maignien et al., 2014) in *Zostera* its composition is highly variable, mirroring their adjacent coastal seawater in eelgrass (Fahimipour et al., 2017). Despite the deep modifications of the leaf physiology and morphology, our Leaf-FISH method successfully generated images of the natural occurring microbial community on eelgrass leaves. Bacteria, labeled with a mixture of probe EUB338 I, II, and III conjugated with Rhodamine Red, were easily observable in the orthoprojections or 3-D images generated of the leaf surface (Figure 2). As expected, bacterial density in this submerged marine plant was much higher than in the terrestrial *Arabidopsis*.

#### Suitability of Leaf-FISH method for bacterial taxa identification on leaf surfaces

To assess the specificity of our protocol we generated a simplified bacterial community by inoculating axenic leaves of *in vitro* grown *Arabidopsis* with a known mixture of some of the most frequently reported phyllosphere bacterial taxa (*Sphingomonas, Pseudomonas*, and *Methylobacterium*) (Vorholt, 2012). Probe testing was performed on pure cultures. Cells (visualized with the DNA stain DAPI) successfully hybridized with the general probe EUB338 I, II, and III (Figures S3A,B). Efficient simultaneous hybridization on filters using general probe EUB338 I, II, and III and genus-specific probes was also achieved for *Methylobacterium* and *Pseudomonas* cultures (mybm-1388 and PSE227, respectively). FISH experiments performed on mixed cultures on filters and on leaves certified the None of our taxon-specific probes showed significant cross hybridization to unintended targets. High hybridization efficiency (Figures S3A,B) and specificity of the selected FISH probes was observed in filters and *in planta* (Figures S3B–D). Spectral unmixing analysis was needed whenever *Pseudomonas* was present, as the specific strain used in this study was characterized by high autofluorescence (see Video S2 in Supplementary Material).

Our method successfully generates taxonomically informative, tridimensional images for visualization of bacteria inhabiting plant surface and subcuticle spaces (Figure S4) or associated with leaf microstructures (Figure 3). Clear specific fluorescence emission associated with each of the fluorophores conjugated with the set of probes targeting cells of *Methylobacterium, Pseudomonas*, and *Sphingomonas* (Figures 3A–C) was observed even in dense bacterial aggregates (Figures 3D–F). Background-free images (Figure 3A) and/or images showcasing specific taxa (Figure S5) can be generated by removing plant autoflourescence signals during post-capture image processing. Under our experimental conditions, *P. putida* W619 was abundant on the leaf surfaces but also it was frequently found inside stomatal cavities and subcuticle spaces (see Figure S4, Videos S2, S3). So, the ability to generate tridimensional images was essential to gain access to the complete distribution of *P. putida* W619 in the *Arabidopsis* phyllosphere (Figures 3A–C).

The ability to manipulate the background signal associated with plant autofluorescence and the strong hybridization signal of taxa-specific probes opens the door to multiple imaging approaches, such as generation of composite images across the irregular surface of leaves. We have generated tridimensional tiled-images to capture bacterial distribution across large areas of the leaf surface, for example 4 × 4 tiles for a total imaged area of 340 × 340 μm (Figure 4A), while simultaneously providing enough resolution to single out rare taxa in a structurally informed context even in a “mock” microbial communities overwhelmingly dominated by a single species (Figure 4B). Post-capture image processing removing plant associated background signal (Figure 4C) generates images suitable for automatic image processing such as standard particle counting analysis as implemented in Fiji (Schindelin et al., 2012; Figure 4D). No discrepancies were observed between cell density estimates calculated by visual counting or by automatic cell counting programs. Additional taxa-specific automatized analysis can be easily performed using different combinations of the layers generated by each probe emission patterns (as those shown in Supplementary Figure S5).

### Practical applications: colonization preferences of methylobacterium sp. on the *Arabidopsis* phyllosphere

Under *in vitro* conditions, we explored the predictive power of genetic similarity and shared physiological traits to infer specific microhabitat preferences of bacteria isolated from the *Arabidopsis* phyllosphere. We paired *Methylobacterium* B5A with another closely related PPFM phyllosphere colonizer, *M. extorquens* PA1 (95% identity to B5A based on 16S rRNA gene; Supplementary Figure S6). PA1 was originally isolated from *Arabidopsis* by Knief et al. (2010a) and the strain we used had been under continuous laboratory culture for many generations (gift of Dr. Chris Marx). Sanger sequencing of the 16S rRNA gene and BLAST analysis against the NCBI database identified our isolate B5A as closely related to *Methylobacterium adhaesivum* (Tani et al., 2012a) (strain 91b) and to the *Arabidopsis* isolate *Methylobacterium* 256 (Knief et al., 2010a) (>99% sequence identity in both cases; Supplementary Figure S7). *Methylobacterium* B5A and PA1 colonize plant leaves under *in vitro* conditions in high numbers from seed inoculation (confirmed by FISH detection). No changes in growth patterns, plant shape or bolting times were observed between inoculated plants and axenic controls.

Besides phylogenetic relationships, we tested for other commonalities such as ability to produce phytohormones. Indol compounds (≥0.25 mg ml^−1^, Salkowski assay) were detected in each isolate in every culturing condition (MM or LB medium) as early as after 3 days in culture and increased along the course of the experiment. After 10 days in culture in MM, no significant differences were detected in indol production (PA1, 0.7–1.75 mg ml^−1^, average 1.1 mg ml^−1^; B5A: 1.4–2.1 mg ml^−1^, average 1.6 mg ml^−1^). Similar results were obtained for cells cultured in LB medium. Biogenic activity of the indol compounds was corroborated using an *in vivo* assay. Two weeks post-inoculation, *Arabidopsis* excised leaves displayed effects typically associated with auxin exposure, such as cellular dedifferentiation and microcalli formation followed by formation of adventitious roots (Supplementary Figure S7). *Arabidopsis* leaves treated with synthetic IAA or inoculated with any of the *Methylobacterium* strains displayed significant higher induced calli/root formation (positive control 1mg/l of IAA: mean 65.9 st.d. 6.2%; PA1: 30.8 ± 6.8%; B5A: 43.9 ± 7.3%; *p* ≤ 0.05 by Kruskal-Wallis/Mann-Whitney) than expected by spontaneous events (negative control: 5.4 ± 2.7%). The stronger response observed in the positive control may be related to the fact that the synthetic auxin is immediately available in the medium to the freshly excised leaves while in the case of phytohormone released by the bacteria, we expect the initial concentration to be low and build up as bacterial density on leaves increases.

The microhabitat preferences these two closely-related, hormone producers, successful colonizers of *Arabidopsis* phyllosphere strains of *Methylobacterium* was examined by imaging the distribution of the microorganisms on leaf surfaces. We imaged leaves from multiple plants from two independently grown cohorts of plants for isolate, single inoculated with PA1 or B5A. The abaxial surface was selected for routine analysis. Visualization was carried out using FISH using probes EUB338 and mybm-1388. Association of bacteria to plant microstructures was qualitatively scored regarding their presence inside the stomata opening and/or cavity, leaf surface, other microhabitats (e.g., side border of the leaf, trichomes) or combination of any of the previous categories. Images were captured in the basal most, mid and apex part of each of the leaves. A total of 50 microscopy fields positive for bacterial presence were examined for *M. extorquens* PA1 and 38 for *M. adhaesivum* B5A. *Methylobacterium extorquens* PA1 was localized in the stomatic area in 78.9 ± 13.10% of the images (Figures 5A–C). In 7.5 ± 10.7% of these cases, PA1 cells were observed simultaneously on leaf surface and stomata areas (Figure 5C). For *Methylobacterium adhaesivum* B5A, simultaneous co-occurrence was 23.8 ± 1.6%. B5A cells were observed exclusively in the stomata area only in 9.9 ± 5.2% of the microscopy. B5A showed completely different microhabitat preferences to that in PA1 and was frequently localized over the leaf cuticle (in over 66.2 ± 3.6% of the fields examined, Figures 5D,E). B5A was usually associated with cracks or grooves although associations to other microstructures such as trichomes (Figure 5F) were also recorded. Under our culturing conditions, the analysis of tridimensional images indicates little or no subcuticular colonization by B5A or PA1. B5A was preferentially found in surface areas (Figure 5E, white arrows) while PA1 cells were confined inside the stomata cavity (Figure 5B, white arrows).

## Discussion

Imaging microbial communities is used extensively to investigate the complex relationships between plants and microorganisms in the rhizosphere (reviewed in Cardinale and Berg, 2015) but to date, few studies have focused on imaging bacteria on the aerial parts of plants. Here, we propose a robust and easily transferable method for simultaneous visualization of multiple bacteria taxa on plant surfaces. By combining leaf pretreatment for pigment removal, fluorescent *in situ* hybridization, and confocal laser scanning microscopy (CLSM), Leaf-FISH method achieves simultaneous visualization of several bacterial taxa at the genus level on a single field of view directly on plant surfaces. Visualization of phyllosphere bacteria on a structural informed context opens new ways to gather information about bacterial colonization strategies, microhabitat preferences, microbial interactions and the relationship of the bacterial community and the plant host. Our method contains four major innovations which enable improved imaging of bacterial communities on leaves. First, our preparation protocol greatly reduces plant autoflouresence, enabling clear visualization of plant microstructures. Second, the use of spectral deconvolution enables reconstruction of 3-D structures below the leaf surface with confocal laser scanning microscopy (CLSM). Third, we are able to generate large 3-D composite images of the leaf surface without compromising resolution. Finally, we demonstrate that Leaf-FISH method is suitable for bacterial taxonomical identification on leaf surfaces with the use of multiple fluorescently labeled oligonucleotide probes.

### Technical advances in Leaf-FISH

Traditionally, plant autofluorescence has been considered a limiting factor for microscopical analysis of bacteria in the phyllosphere (Shinkai and Kobayashi, 2007; Piccolo et al., 2010; Remus-Emsermann et al., 2014) as the extremely high background noise generated by the intrinsic autofluorescence of plant tissues blurs targeted signals. Our method uses an ethanol-mediated pigment removal step to reduce the plant autofluorescence while retaining enough signal to accurately recreate leaf microstructures using CLSM. Our method overcomes the detrimental effects of plant autofluorescence and use them in our favor to address the bacterial-plant relationships in a contextualized space. Leaf autofluorescence signal(s) independently acquired and treated as another fluorophore(s), generates fully editable layers that can be modified or even removed during post-capturing processing.

One elegant and successful approach to bypass plant autofluorescence and explore the distribution of bacterial communities in plant tissues is TAPE-FISH (Bisha and Brehm-Stecher, 2009; Remus-Emsermann et al., 2014) which consists in the transference of surface microorganisms to an adhesive tape. This step substitutes the complex surface of plant leaves with a 2D filter-like surface much more suitable for routine FISH experiments. However, *in planta* tridimensional phyllosphere images, such as those generated by Leaf-FISH, add structural context to the study of plant-bacteria interactions, showing how often more than a single factor may affect cell distribution. CLSM generates consecutive optical slides by acquiring fluorescence emitted by the target probes and plant tissues from each focal plane and then integrates these independently captured Z-stack images into a single tridimensional image. Leaf surfaces are irregular surfaces that can show drastic topographical changes in relative small distances, such as those shown in Figures 1D–F, where a vertical 6–7 μm drop can be observed over a distance of 15–20 μm. In some cases, bacteria were observed on also elevated areas (surface “peaks”) instead of in sheltered “valley” areas. However, this unexpected result was explained by adding structural context to the image (Figure 1E), showing deep cuticle irregularities in the area accumulating the higher bacterial densities.

Using CLSM we overcame the difficulty in maintaining several areas of the leaf in focus simultaneously, by generating large 3-D composite images (Figure 4; 340 × 340 μm^2^) without compromising the resolution at the single cell level. The ability to image large areas of the leaf surface can help to obtain accurate global or taxa-specific cell densities of naturally occurring microorganisms in the phyllosphere, avoiding biases due to patchy bacterial distributions. However, hybridization techniques may introduce other biases. Remus-Emsermann et al. (2014) reported that specific groups of phyllosphere bacteria characterized by the presence of infrared autofluorescence failed to display any hybridization signal using a combination of TAPE-FISH and epifluorescence microscopy. In our analysis, we did not observe any erratic hybridization behavior of bacterial strains characterized by autofluorescence (e.g., *Pseudomonas* or *Methylobacterium* B5A, PA1) in pure cultures or *in planta* (e.g., Figures S3A,B). Although not covered in the present manuscript, it is also important to consider that visualization of Gram-positive taxa might require modifications of the fixation step (Thurnheer et al., 2004). Finally, hybridization-based techniques include multiple steps that especially when performed on tissues not coated in resins or agar, could potentially alter the spatial distribution of bacteria on the phyllosphere. Our method, in addition, requires a dehydration gradient in ethanol to remove autofluorescence. To stablish the role of our method in a potential bacterial redistribution events, further experiments especially designed to tackle this question should be performed. Next steps in development of the Leaf-FISH method should include the comparison of distribution patterns detected through different visualization methods, such as direct visualization of cells expressing fluorescence proteins vs. Leaf-FISH (see Brandl et al., 2001 for example).

### Implications for phyllosphere microbiology

Imaging methods greatly improve our understanding of phyllosphere colonization processes. Leaf microorganisms can be already present during leaf development or, most frequently, colonize it from environmental sources (Leveau, 2015). For immigrant cells, random events play a large role in determining colonization success as most leaf microhabitats do not support high population numbers (Remus-Emsermann and Leveau, 2010). Under favorable conditions, migrant cells will divide, and the daughter cells will remain together to form aggregates. Microsite-specific variables such as water availability will determine the final population size necessary to trigger quorum sensing mediated responses (Dulla and Lindow, 2008). For some phytopathogens frequently found on plant surfaces, such as *Pseudomonas* sp., the leaf apoplast is the preferred habitat. Epiphytic and apoplastic life-styles are characterized by different nutritional sources and stresses characteristic of each microhabitat, each of them requiring differential metabolic responses (Yu et al., 2013). Tridimensional images generated by Leaf-FISH method can shed light on colonization of these compartments. Transversal analysis of tridimensional images of naturally occurring bacteria confirm that some taxa rarely colonized subcuticle spaces (Figures 5D–F) while others form aggregates that frequently behave as a unit along the cuticle and subcuticular compartments such as stomata chambers (Figures 1A, 5A–C, Figure S4, Videos S2, S3). Using *Pseudomonas* autofluorescence (Video S2 in Material) and FISH with taxon-specific probes (Video S3 in Supplementary Material), we showed that our method is suitable for identification of *Pseudomonas* cells in both the cuticle and immediate subcuticular areas. These parts of the leaf are not accessible by surface imaging methods, such as TAPE-FISH. Therefore, tridimensional imaging is a considerable advance to study the natural continuous distribution of bacteria in the phyllosphere, including the upper layers of apoplast or stomata chambers, and record different stages of colonization without sectioning.

### Case study: methylobacterial colonization of *Arabidopsis*

Simultaneous visualization of cuticle, subcuticular areas, stomata chambers and other microhabitats provides highly informative data on specific microhabitat preferences and colonization strategies of phyllosphere bacteria. To illustrate this point, we selected two closely related pink-pigmented facultative methylotrophic (PPFM) bacteria to carry out controlled colonization experiments of the *Arabidopsis* phyllosphere. *Methylobacterium* use methanol as a source of carbon and energy (Delmotte et al., 2009) and successfully colonize the phyllosphere of *Arabidopsis*. PPFM are known producers of phytohormones such as IAA (Omer et al., 2004) and their presence has been reported as beneficial for protonemata development in mosses (Hornschuh et al., 2002), plant growth (Madhaiyan et al., 2004; Tani et al., 2012b) and increased systemic resistance (Madhaiyan et al., 2006). In turn, plant associated bacteria may benefit from manipulating plant hormonal levels (Faure et al., 2008) to increase their fitness by locally modifying phyllosphere micro-habitats (Lindow and Brandl, 2003). We demonstrated that *Methylobacterium extorquens* PA1, a well-characterized bacterium isolated from the *Arabidopsis* phyllosphere (Knief et al., 2010a), and *Methylobacterium adhaesivum* B5A, newly isolated in this study, produce IAA active compounds in pure culture and in co-culture with plant leaves. Both *Methylobacterium adhaesivum* and *M. extorquens* are efficient phyllosphere colonizers as shown here and elsewhere (Knief et al., 2010a; Verginer et al., 2010; Wellner et al., 2011). Differential colonization strategies have been long suspected for *Arabidopsis-* associated *Methylobacterium*. (Knief et al., 2010a) proposed that the higher densities reported for *Arabidopsis* plants inoculated with different *Methylobacterium* strains than those in plants inoculated with a single strain could be related to differential niche occupation. Our results confirm taxa-specific preferential microhabitats in *Methylobacterium*, with B5A inhabiting cuticle and occasionally on trichomes (Figure 5F) and *M. extorquens* PA1 (Figures 5A–C) was almost exclusively found in the stomatal chamber.

Determining microhabitat preferences has important practical implications for understanding, and potentially controlling, pathogen infection through the leaf surface as well as the spread of human pathogens via colonization of the leaf surface (Rastogi et al., 2013; Erlacher et al., 2015). Many human pathogens with significant public health implications, including *Salmonella, Shigella* and *E. coli* O157:H7, attach or colonize to plant leaves (reviewed in Berger et al., 2010). Some preferentially colonize specific leaf microhabitats, such as stomatal cavities, that can provide protection during chemical decontamination (Saldaña et al., 2011). Positive and negative correlations between pathogen presence and community composition have been reported (Rastogi et al., 2012), suggesting the potential use of commensal bacteria to limit colonization by plant and human pathogens. (Innerebner et al., 2011) reported that the ability of plant-protective *Sphingomonas* strains to achieve high densities in the phyllosphere was not enough to diminish *Pseudomonas* infectivity, indicating that other factors, perhaps derived from specialized close-range interactions, might play a determinant effect in phytoprotective behaviors. Leaf-FISH is suitable for visualization of has the potential to enable a deeper exploration of taxa-specific colonization strategies, distribution, and commensal and pathogenic bacteria interactions on leaf surfaces to understand the spatial preferences that may play a significant role in pathogen-commensal dynamics.

We present here a robust imaging protocol suitable for identification at the genus level the bacteria present in the cuticle and subcuticlar areas of the plant leaves. The transferability Leaf-FISH method to other plant systems together with the suitability for combining large numbers of probes opens the door to the study of microbial interactions on leaf surfaces at the strain level. In addition, the ability to create high resolution tridimensional images of the plant surface will be undoubtedly useful to gain a deeper understanding of microorganism-plant interactions.

## Author contributions

EP and SS designed this study and drafted the manuscript. EP grew *in vitro* plants, isolated and identified the phyllosphere bacteria, and generated the epifluorescence and CLSM images. EP and SS analyzed the data and defined the imaging protocol. Both authors read and approved the final manuscript.

### Conflict of interest statement

The authors declare that the research was conducted in the absence of any commercial or financial relationships that could be construed as a potential conflict of interest.
