# Supplementary material for: Leaf-FISH: Microscale Imaging of Bacterial Taxa on Phyllosphere
Source: Front Microbiol. 2018 Jan 9;8:2669. doi: 10.3389/fmicb.2017.02669 (PMC5767230; doi:10.3389/fmicb.2017.02669)
Supplement: Supplementary file 1 [file DataSheet1.doc]

Supplementary Material

Leaf-FISH: Microscale imaging of bacterial taxa on phyllosphere .

Elena L. Peredo*^1,2^, Sheri L. Simmons^1^.

^1^ Josephine Bay Paul Center, Marine Biological Laboratory, Woods Hole, MA, USA.

*** Correspondence:** Corresponding Author: Josephine Bay Paul Center, Marine Biological Laboratory, Woods Hole, MA, USA. [elperedo@mbl.edu](mailto:elperedo@mbl.edu)

^2^ **Present address:** The Ecosystems Center, Marine Biological Laboratory, Woods Hole, MA, USA.

1. **Material and Methods**
   1. **Quantification of bacterial losses during leaf fixation method.**

Protocol-related cell losses were estimated in leaves of *Arabidopsis* plants germinated in sterile conditions and colonized in a greenhouse environment, which results in a diverse phyllosphere bacterial community (Maignien et al., 2014). The total number of cells washed away from the leaf surfaces was quantified by calculating the bacteria present in buffers and solutions used during each step of the fixation and dehydration series. To facilitate comparison, we express losses as the percentage of cells removed of total bacteria estimated for leaf surfaces per square centimeter. The average leaf area of greenhouse grown plants was 1.44 cm^2^ (0.72 cm^2^ ± 0.36 each leaf blade) calculated (n=6) with ImageJ 1.47 (<http://imagej.nih.gov/ij/>).

A mixture of leaves obtained from different plants from the same greenhouse tray were pooled and subject to our fixation and pigment removal protocol (three replicas, each n=12 leaves). The total volume of liquids produced during fixation and ethanol removing steps for each replica was filtered through a 0.22 μm filter (GTTP, Millipore, Billerica, MA, USA). The total number of bacteria in each filter, and therefore washed during ethanol removal in each of the replicas, was calculated extrapolating the average number of bacteria per 100 X 100 μm field of view to the total filter area. Sections of the filters were fixed and subject to a standard *in situ* hybridization protocol using general bacterial probe EUB338 I, II, and III, covered in ProLong Gold with DAPI and stored overnight in the dark. Images were generated using an epifluorescence microscope (Zeiss Axioskop2). For the three replicas, the average number of cells per field of view were 2.66±1.44, 1.73±1.33, and 1.66±1.83 (see Fig. S1D and E for examples). We calculate that as average, 9.54 x 10^4^ cells washed in total which translates to ~7.95x 10^3^ cells per each of the twelve leaves polled for Leaf-FISH. Using a conservative estimation of bacteria density of 10^6^ cells cm^-2^ (Vorholt, 2012), our protocol produced average percentage of losses of 0.55% of the total bacteria present on the phyllosphere for greenhouse material.

We calculated the average cell density on leaves (n = 4 leaves, 10 random fields per leaf) of greenhouse grown *Arabidopsis* using Leaf-FISH. Each field was a tridimensional image of the leaf surface formed by 10-15 images obtained one micron apart along the Z-axis (85 X 85 X 10-15 µm^2^). The estimated cell density was 2.7 x 10^5^ cells cm^-2^, consistent with estimates obtained with qPCR (Maignien et al., 2014). Using this lower cell density defined for our experimental system, we re-estimated the cell loss caused by the fixation and ethanol removal protocol in 2.7%. We consider that cell losses caused by our protocol (<5%) are acceptable. However, we strongly encourage the evaluation of cell loss during the protocol as a matter of course in applications of our method to different plant species.

In a second experiment we determined bacterial surface losses in *in vitro* grown *Arabidopsis* plants, seed-inoculated with known strains of *Methylobacterium,* using the same approach as described before. A mixture of leaves (n= 15) obtained from different plants from the same *in vitro* microbox were pooled and subject to our fixation and pigment removal protocol. The average number of cells removed from surface was calculated in 9.86 x 10^3^ per leaf or 1.23 x 10^4^ cells cm^-2^ of leaf surface *in vitro* plants inoculated with *Methylobacterium* *extorquens* PA1 and, 6.92 x 10^3^ per leaf or 8.65 x 10^3^ cells cm^-2^ in the case of those plants inoculated with *M. adhesivum* B5A. Using 10^6^ cells cm^-2^ as general estimate of bacterial density, the bacterial losses are 1.23% in the case of PA1 and 0.86% in B5A. The higher cell losses in the *in vitro* material is not an unexpected as cell density is dependent on environmental humidity which is higher in the *in vitro* system than in most open systems. Also, the sheltered conditions inside the microboxes might result in looser attachment of the microorganisms to their host.

***De novo* isolation of bacteria from the *Arabidopsis* phyllosphere.**

Bacterial isolates were obtained from a single mature-senescent (after flowering) *Arabidopsis thaliana* plant cvs Columbia (Col-0) (Lehle Seeds, Round Rock, TX, USA) grown hydroponically under greenhouse conditions (Falmouth Technology Park, Falmouth, USA) as described in (Maignien et al., 2014). Floral stalks and roots were excised and discarded beforehand. The plant material was manually ground in 50 ml of filtered, autoclaved, distilled water. The macerate was recovered using a sterile syringe and plant debris was removed by filtering through a 180 µm nylon net filter (NY8H, Millipore, Billerica, MA, USA) mounted on an autoclaved Swinnex filter holder (Millipore, Billerica, MA, USA). 250 μl of serial dilutions of the macerate (10^-1^, 10^-2^, and 10^-3^) were plated in square petri dishes. Two different solid growth media (1.5% agar) were used for bacterial culturing: a general low salt rich medium (LB) (Luria Broth, US Biological, Swampscott, MA, USA) and a selective salt medium with 120 mM methanol (MM) (Delaney *et al*., 2013). To prevent fungal growth, media were supplemented with 50 mg l^-1^ of cycloheximide (Sigma-Aldrich, St. Louis, MO, USA). Plates were kept at room temperature for 5-7 days. Colonies with different morphologies (color, size, shape) from both media were re-streaked at least twice or until pure cultures were achieved (total 128 isolates; 71 in LB, 57 in MM). The identity of each isolate was assessed with amplification of the 16S rRNA gene with direct PCR of individual colonies using primers 27F-YM and 1492R (Frank et al., 2008). Fragments were purified using Ultraclean PCR Clean-up kit (MoBio laboratories, Carlsbad, CA, USA) followed by Sanger sequencing. Sequences were compared to the Genbank 16S database using BLAST. 27% of the isolates were identified as *Methylobacterium*. Other frequent bacteria were *Arthrobacter*, *Bacillus* and *Ralstonia*. We also isolated other taxa typically found in the phyllosphere, including *Sphingomonas*, *Mesorhizobium*, and *Rhizobium.* We screened the colonization efficiency of several phyllosphere-derived bacterial strains (*Ralstonia*, *Sphingomonas*, *Methylobacterium,* and *Arthrobacter*) and their effects on plant phenotype. Out of all the isolated characterized, *Methylobacterium* B5A was selected for further analysis because of its ability to colonize plant leaves in high numbers under *in vitro conditions* (confirmed by FISH detection). No changes in growth patterns, plant shape or bolting times were observed between plants inoculated with B5A and axenic controls. Further characterization of the isolated *Methylobacterium* strains was performed by analysis of the ITS region using primers 1319F-G20 and 45R following published protocols (Knief et al., 2008). Phylogenetic placement of *Methylobacterium* 16S and ITS sequences was obtained by generating Maximum Likehood trees. Sequences were aligned to *Methylobacterium* sequences available in Genbank using Muscle as implemented in Geneious 5.9 (Biomatters, http://www.geneious.com/). ML trees (Fig. S6) were run in RAxML (Stamatakis, 2006) (10 runs, 1000 bootstrap repetitions) and visualized with Figtree (http://tree.bio.ed.ac.uk/software/figtree/). Newly generated sequences were accessioned in GenBank (KT949348-KT949393). Genbank accession numbers of additional 16S sequences not generated in this study: FN868943, FN868933, AB175633, FN868947, DQ471331, FN868949, AM910539, FN868952, AB252208, FN868934, AB175636, FN868950, FN868935, FN868939, FN868961, FN868957, FN868941, FN868954, AB302928, FN868936, FN868946, AM910535, FN868944, AB698720.

- 1. **References**

Frank, J., Reich, C. I., Sharma, S., Weisbaum, J. S., Wilson, B., and Olsen, G. J. (2008). Critical evaluation of two primers commonly used for amplification of bacterial 16S rRNA genes. *Appl. Environ. Microbiol.* 74, 2461–70. doi:10.1128/AEM.02272-07.

Knief, C., Frances, L., Cantet, F., and Vorholt, J. A. (2008). Cultivation-independent characterization of *Methylobacterium* populations in the plant phyllosphere by automated ribosomal intergenic spacer analysis. *Appl. Environ. Microbiol.* 74, 2218–28. doi:10.1128/AEM.02532-07.

Maignien, L., Deforce, A. E., Chafee, M. E., Eren, A. M., and Simmons, S. L. (2014). Ecological succession and stochastic variation in the assembly of *Arabidopsis thaliana* phyllosphere communities. *MBio* 5, e00682–13. doi:10.1128/mBio.00682–13. doi:10.1128/mBio.00682-13.

Vorholt, J. A. (2012). Microbial life in the phyllosphere. *Nat. Rev. Microbiol.* 10, 828–840. doi:10.1038/nrmicro2910.

1. **Supplementary Figures**

Supplementary Figure S1: Visual summary of methods.

Supplementary Figure S2: Bacteria on *Arabidopsis* leaves from in vitro grown plants seed-inoculated with *Methylobacterium* imaged with routine microscope for transmitted light and incident light fluorescence (Zeiss Axioskop2).

Supplementary Figure S3: Examples of probe specificity testing.

Supplementary Figure S4: *Pseudomonas* on the leaf surface and immediate subcuticle area.

Supplementary Figure S5: Image decomposition of Figure 3A-C.

Supplementary Figure S6: Phylogenetic placement of newly isolate *Methylobacterium* B5A based in 16S.

Supplementary Figure S7: Adventitious root development triggered by the presence of *Methylobacterium*.

Supplementary Video S1: Bacteria visualization using (1) fluorescence from the EUB338 probe, (2) transmitted light captured during confocal scanning microscopy, and (3) overlap both signals.

Supplementary Video S2: *Pseudomonas* inoculated to *Arabidopsis* leaf.

Supplementary Video S3: Bacteria present in *Arabidopsis* leaf (Figure 3A-C).


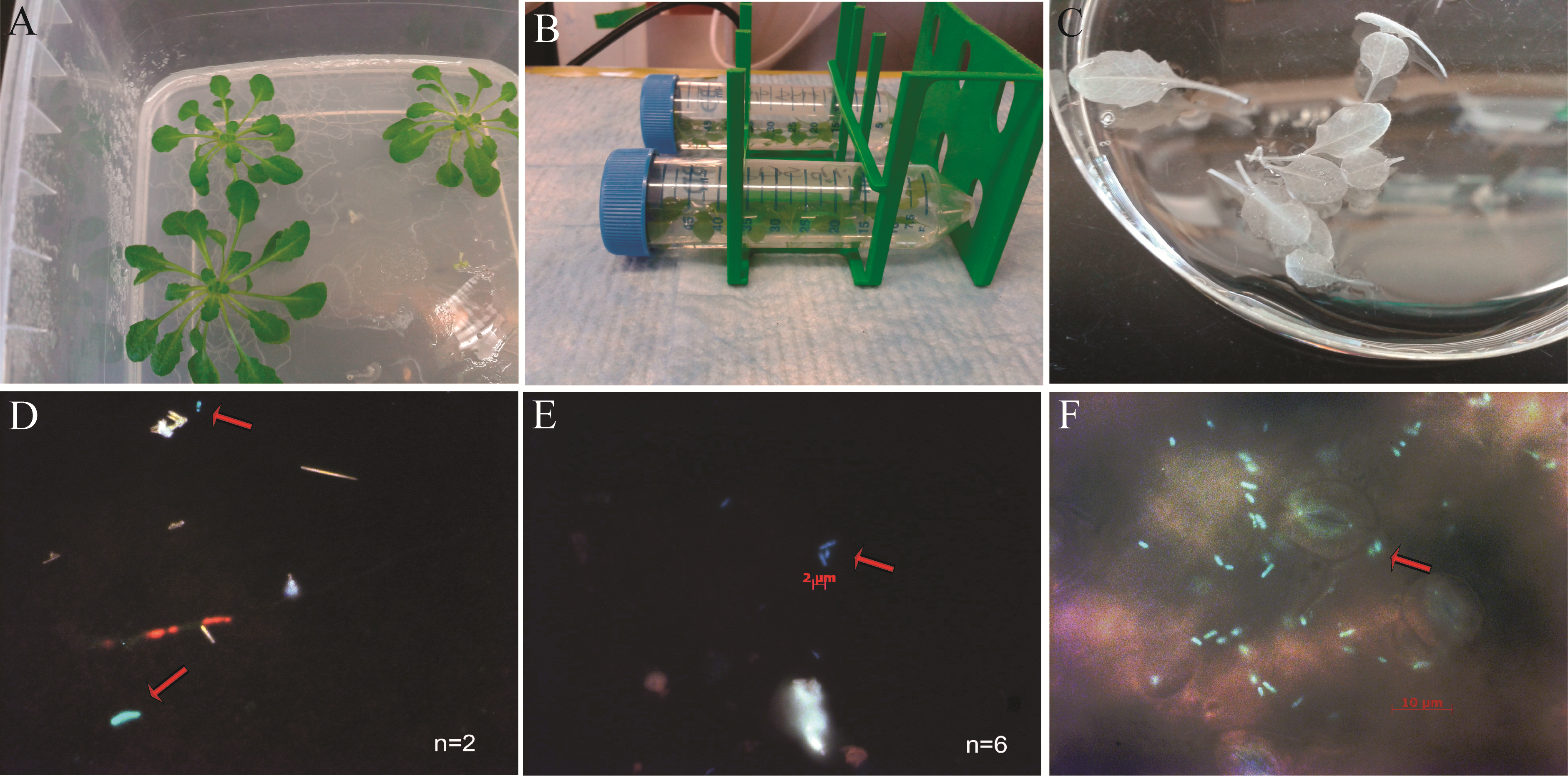


**Supplementary Figure S1:** **Visual summary of methods**. **(A)** 6 week old *Arabidopsis* *in vitro* plants grown in microboxes (0.5 X MS medium with vitamins pH 5.8, 0.7% agar). **(B)** Fixation of 10 leaves for 1 hour in 10 ml of 1 X PBS supplemented with 2% paraformaldehyde. **(C)** Rehydration of leaves after pigment removal protocol. **(D, E)** Representative image of a 100 X field of control filter containing cells washed after the fixation and ethanol treatment. In this case, the cells correspond to twelve leaves from *Arabidopsis* plants growing in our greenhouse. Among other debris collected in the filter, such as dust, two bacterial cells can be observed panel D in bright green (arrows, FISH EUB388 I, II, and III conjugated with Alexa488) and 6 cells in E. **(F)** Image of a 100 X field of an *Arabidopsis* leaf growing in our greenhouse


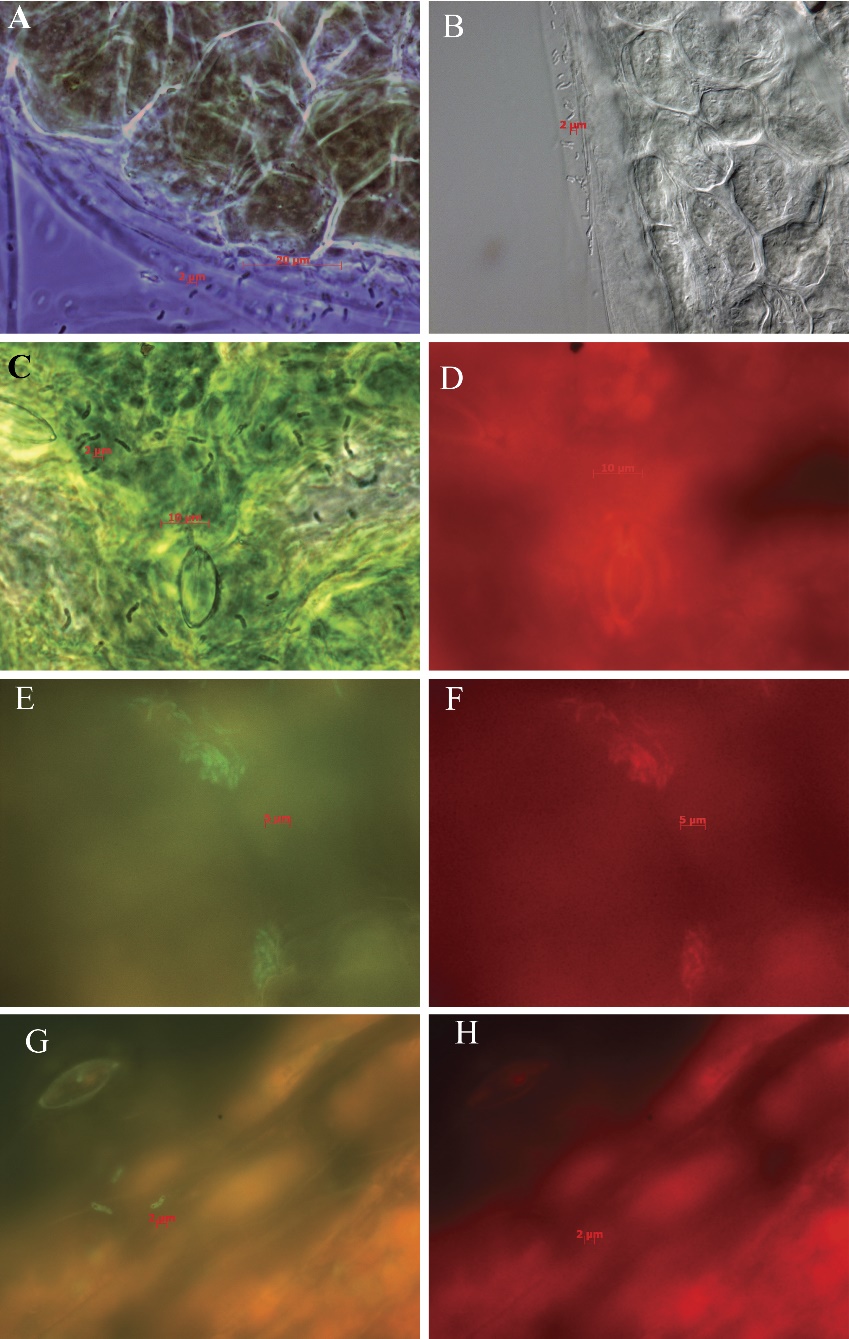


**Supplementary Figure S2: Bacteria on *Arabidopsis* leaves from *in vitro* grown plants seed-inoculated with *Methylobacterium* imaged with routine microscope for transmitted light and incident light fluorescence (Zeiss Axioskop2)** **(A,B)** Control and bleached *Arabidopsis* leaves visualized using transmitted light. Bacterial cells can be observed in the border of the leaf (X63). Note: bacterial cells in image A are loosely attached to plant surface due to a pretreatment step (vacuum treatment) tested while developing the protocol. Vacuum was found to have strong effect in bacterial losses, so was discarded. **(C,D)** Bacteria on surface of control leaf are visible using transmitted light. Bacteria were labelled with EUB388 I, II, and III probe conjugated with Rhodamine, background autofluorescence was too high in the untreated material to visualize the signal of the labeled probe. **(E,H)** Bacteria on *Arabidopsis* *in vitro* leaves treated with pigment removal steps, visualized using fluorescence. **(E,F)** Bacteria were labelled with mybm-1388 and conjugated with Alexa-488 (green) and EUB388 I, II, and III probe conjugated with Rhodamine (red). **(G)** Bacteria only labelled with mybm-1388 conjugated with Alexa-488 (green) so no signal is observed with red filter **(H).** Images C-H were acquired with 100 X objective lens.


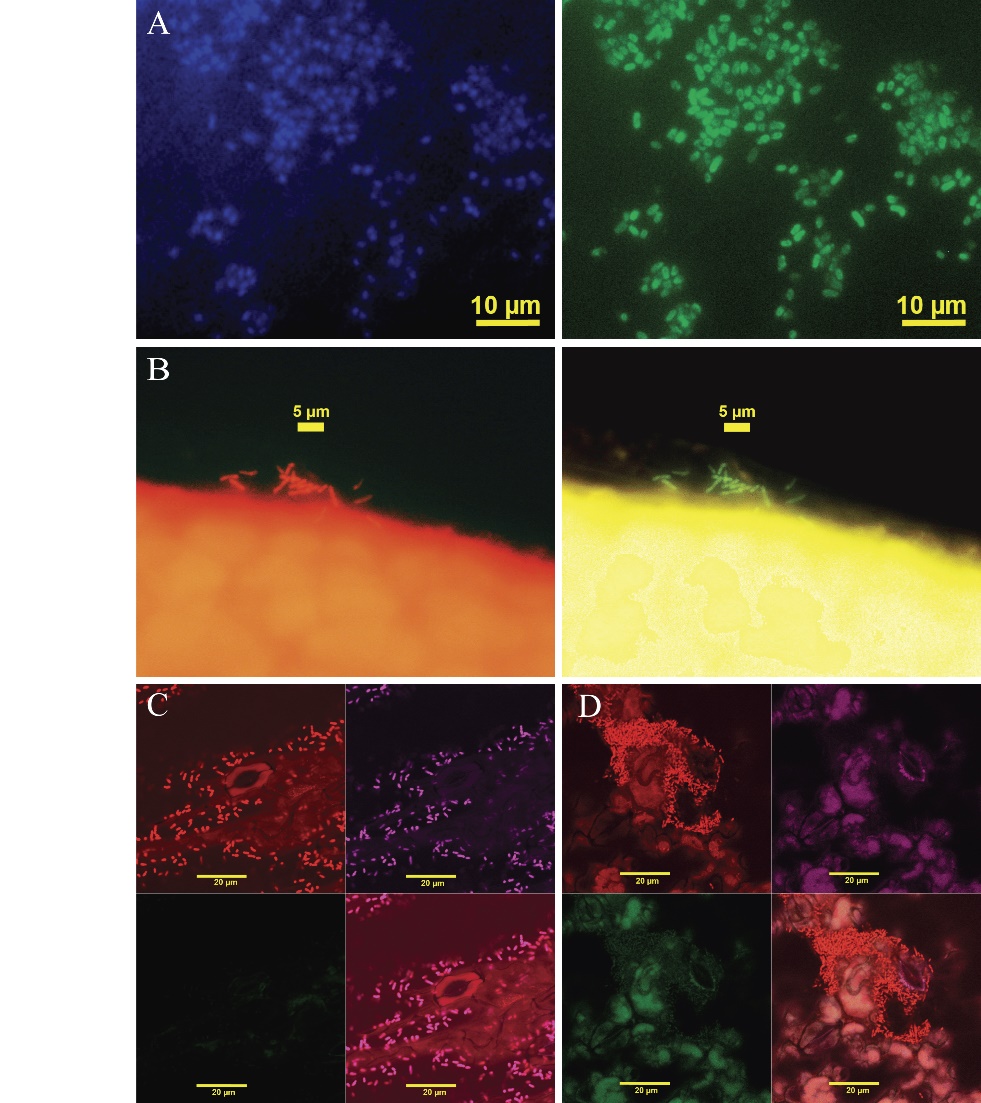


**Supplementary Figure S3:** **Examples of probe specificity testing**. **(A)** On filter: *Methylobacterium* cells visualized using DAPI (blue, left) and hybridized with probe mybm-1388 (Alexa488-right). 100 X lens, routine microscope (Zeiss Axioskop2). **(B)** Bacterial cells on a leaf from plants inoculated with *Methylobacterium* *extorquens* PA1. All cells were simultaneously labeled with EUB388 (red, left) and mybm-1388 (green, right). 100 X lens, routine microscope (Zeiss Axioskop2). **(C,D)** Confocal images (100 X lens, Zeiss LSM780) showing the probe specificity and low crosstalk of the selected fluorophores when used *in situ*, on *Arabidopsis* leaves independently co-cultured with **(C)** *Methylobacterium* *adhaesivum* B5A, or **(D)** *Sphingomonas* sp. Hybridization mix included EUB388 I, II, and III (shown in red), mybm-1388 (pink) and PSE227 (green). **(C)** *M.* *adhaesivum* B5A hybridization signal is observed in the with EUB388 I, II, and III (red) and mybm-1388 (pink). **(D)**. *Sphingomonas,* signal is only detected in EUB388 I, II, and III (red). No fluorescent signal, beside plant associated background, was observed with mybm-1388 or PSE227. In both panels, from top left to bottom right: Rhodamine channel (red, EUB388 I, II, and III); Alexa-488 channel (pink, mybm-1388); Alexa-647 channel (green, PSE227); all channels. To provide a more accurate representation of the behavior of the probes in planta, images in panel C and D were captured in channel mode and not processed using spectral unmixing.


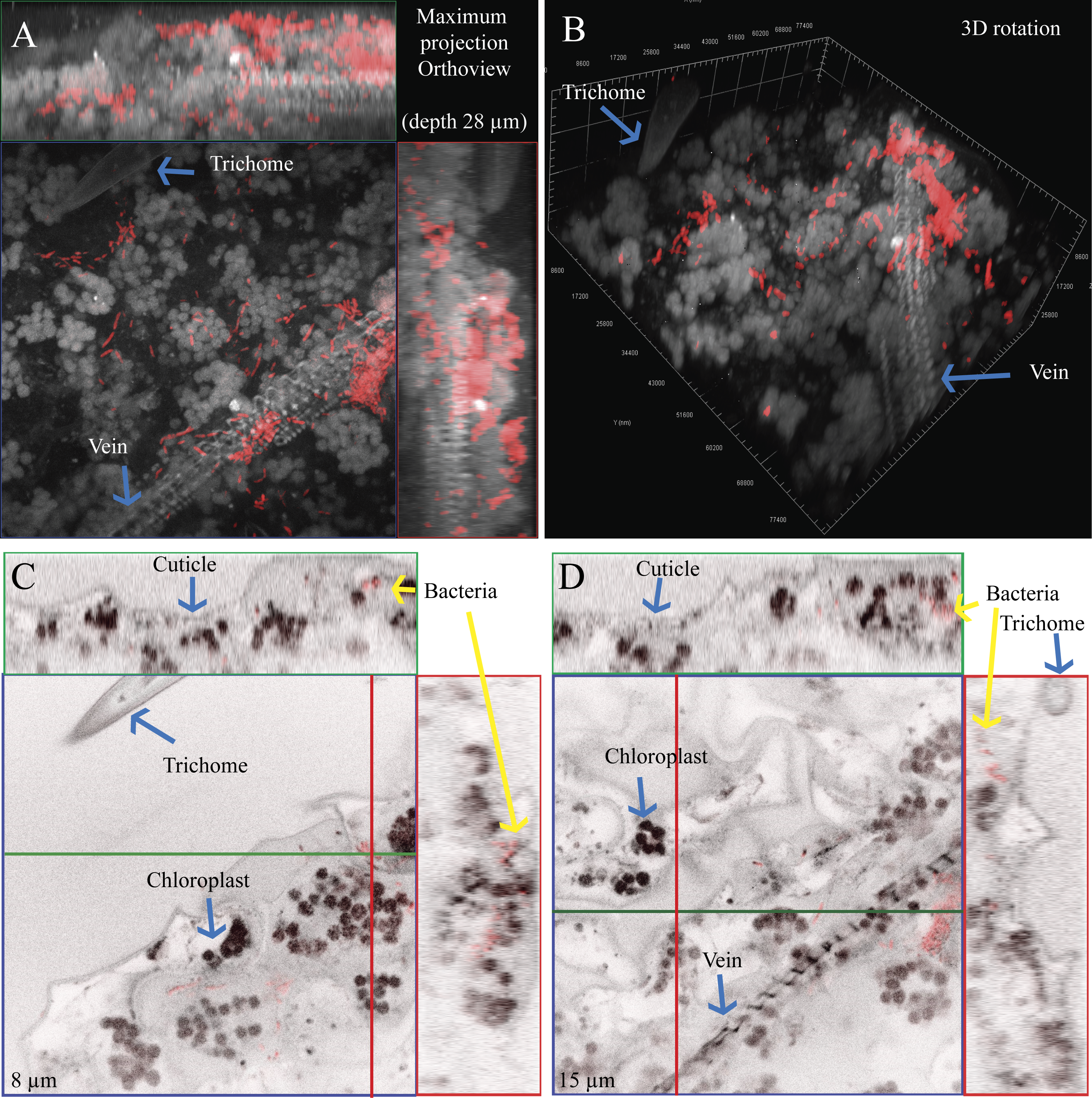


**Supplementary Figure S4:** **Imaging of microbial aggregates on the leaf surface and immediate subcuticle area of *in vitro* grown *Arabidopsis* after co-culturing with *Pseudomonas*.** All confocal images were generated using a 100 X lens, Zeiss LSM780 and processed using spectral unmixing. *Pseudomomas* cells are labeled with EUB338 I, II and III, and are false colored in red. **(A)** Maximum projection orthoview (X, Y, and Z) of Z-stack images, 28 µm depth showing *Pseudomonas* in the subcuticle area. Plant autofluorescence is shown in grey. **(B)** 3D view of same image. (**C** and **D**) Different orthoviews recreated in Fiji from confocal unmixed images. Channel with EUB338 signal and channel accumulating most of the unmixed background signal from plant tissues were independently imported, modified for visualization purposes, and merged to generate orthoviews showcasing surface irregularities and plant structures. Blue arrows signal plant structures, yellow arrows bacteria (in red).


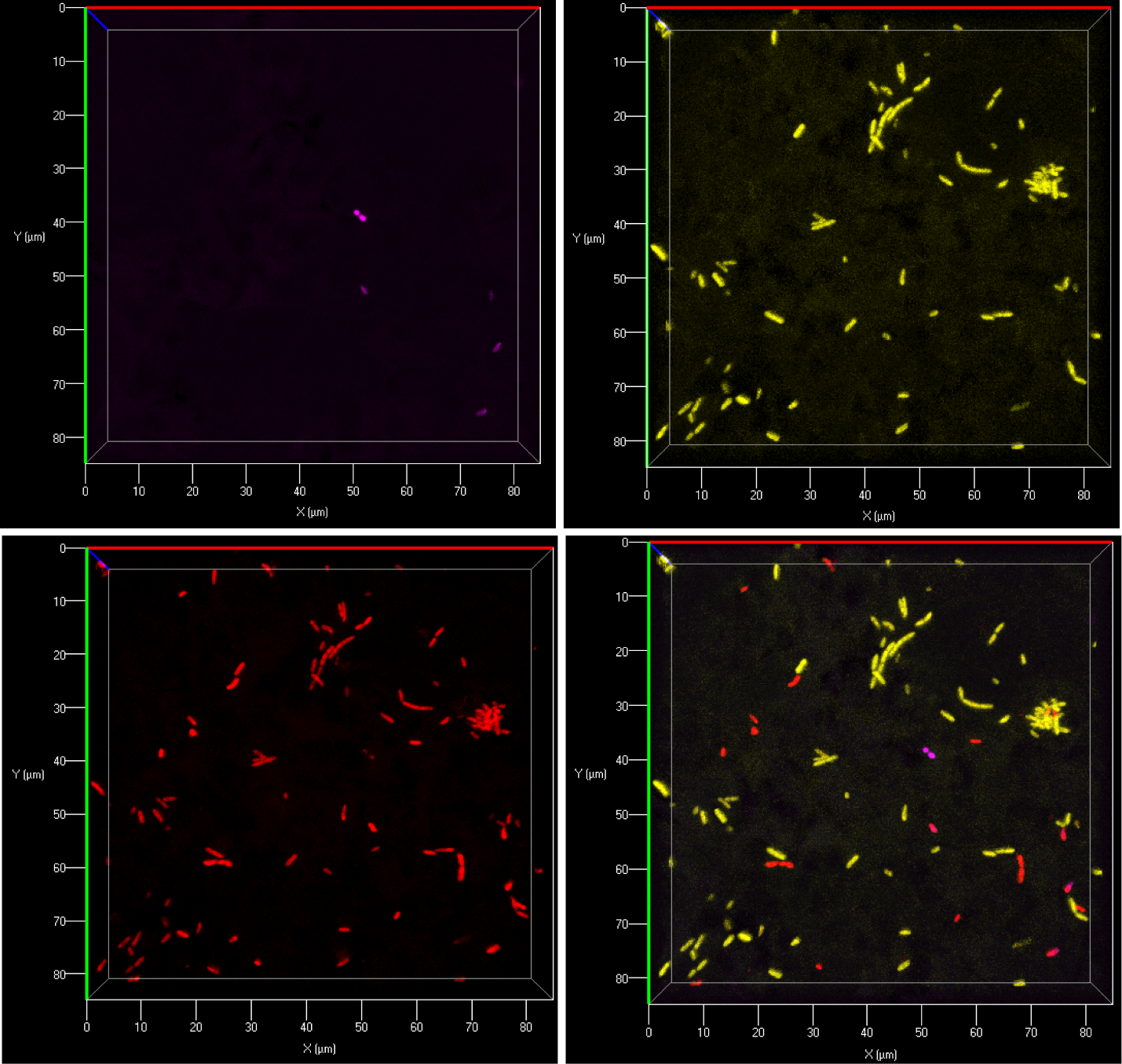


**Supplementary Figure S5: Image decomposition of Figure 3A-C.** (Bacteria on leaf surface of *in vitro* grown *Arabidopsis*). Hybridization mix included probes mybm-1388 (top left, *Methylobacterium* pink), PSE227 (top right, *Pseudomonas* yellow), and EU388 I, II, and III (bottom left, all bacteria red). All probes combined (bottom right, *Methylobacterium* pink, *Pseudomonas* yellow, *Sphingomonas* red). 3-D images generated from Z-stack, 16 confocal images (100 X lens, Zeiss LSM780).


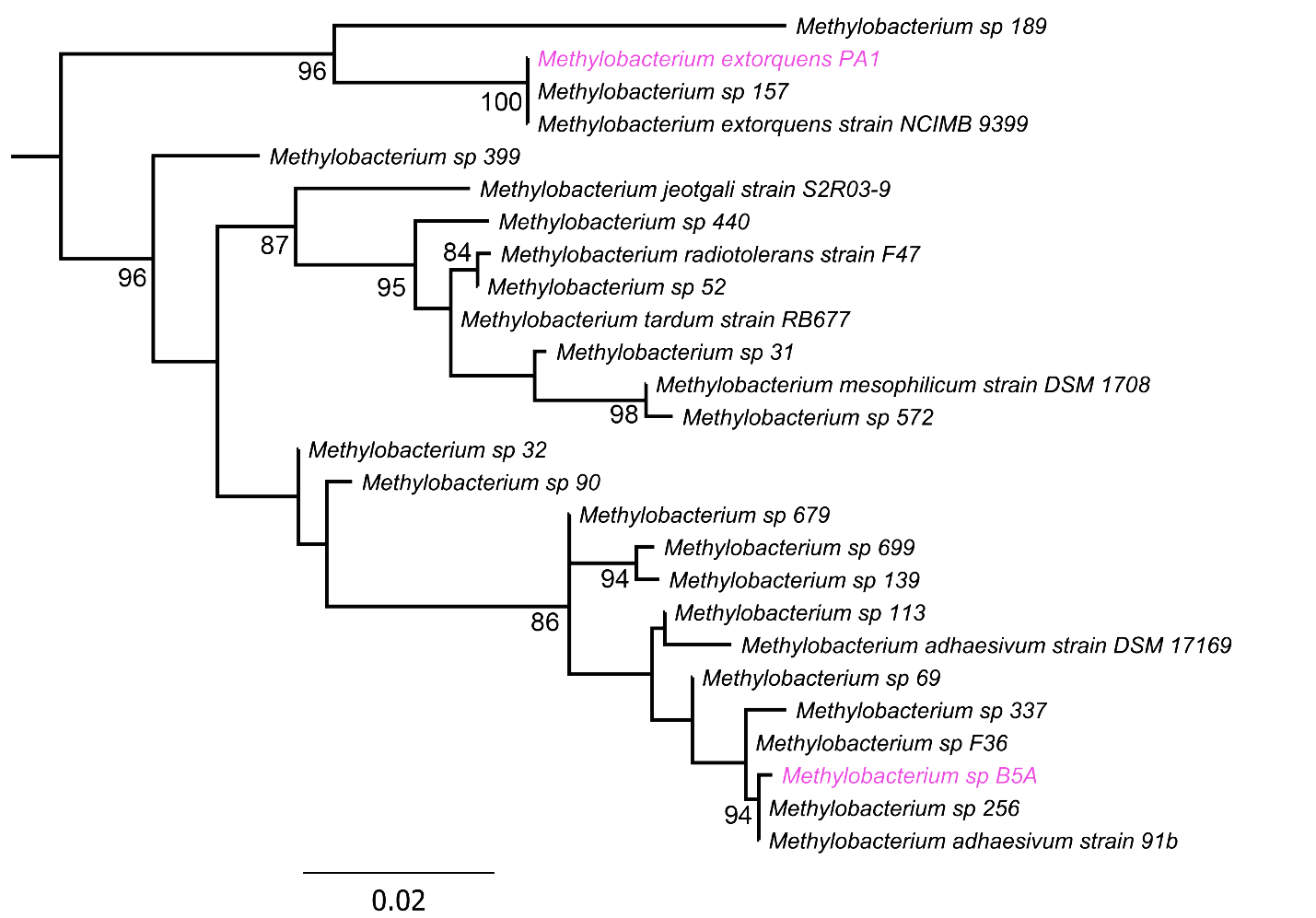


**Supplementary Figure S6:** **Phylogenetic placement of newly isolate *Methylobacterium* B5A based in 16S**. Sequences were aligned to *Methylobacterium* sequences available in Genbank (KT949366, KT949367). Maximum likehood trees were run in RAxML (10 runs, 1000 bootstrap repetitions). *M. adherents* B5A and *M. extorquens* PA1 are shown in pink. Genbank accession numbers of additional 16S sequences not generated in this study: FN868943, FN868933, AB175633, FN868947, DQ471331, FN868949, AM910539, FN868952, AB252208, FN868934, AB175636, FN868950, FN868935, FN868939, FN868961, FN868957, FN868941, FN868954, AB302928, FN868936, FN868946, AM910535, FN868944, AB698720.


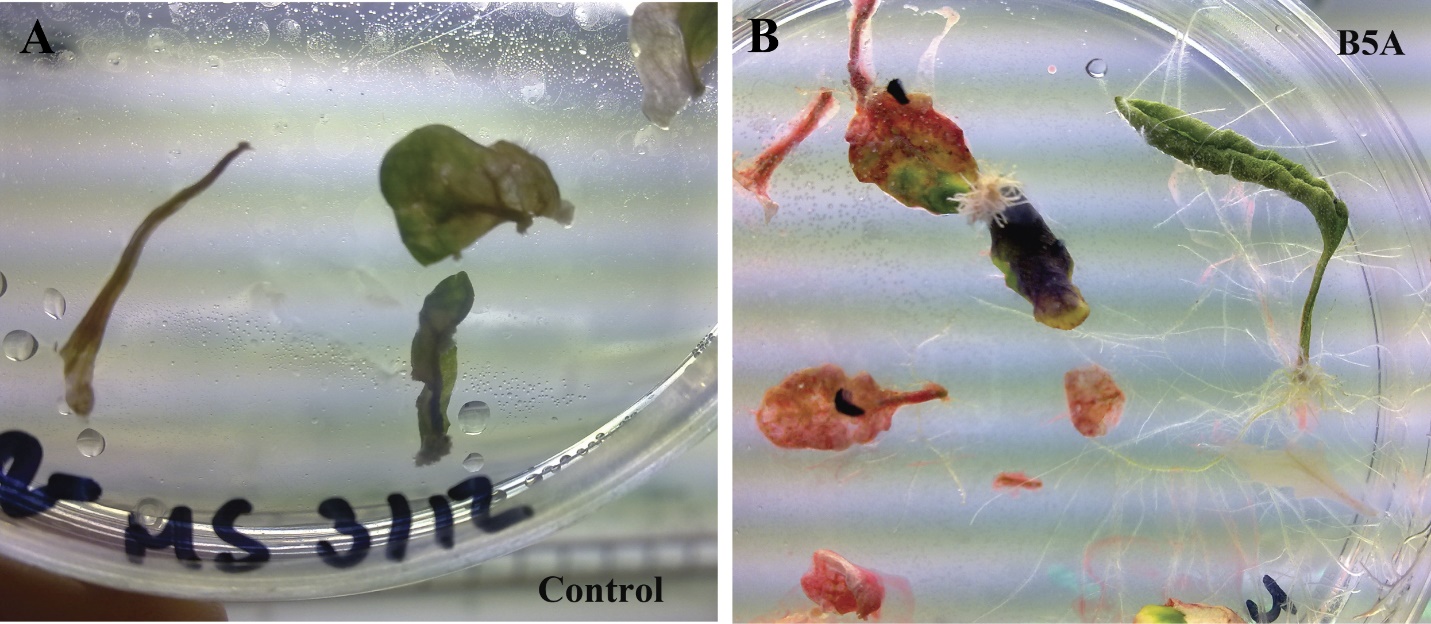


**Supplementary Figure S7:** **Adventitious root development triggered by the presence of *Methylobacterium***. **(A)** Control leaves after 4 weeks. **(B)** *Methylobacterium*-inoculated leaves.
